# Tail-Engineered Phage P2 Enables Delivery of Antimicrobials into Multiple Gut Pathogens

**DOI:** 10.1021/acssynbio.2c00615

**Published:** 2023-02-02

**Authors:** Jidapha Fa-arun, Yang Wei Huan, Elise Darmon, Baojun Wang

**Affiliations:** † College of Chemical and Biological Engineering & ZJU-Hangzhou Global Scientific and Technological Innovation Center, Zhejiang University, Hangzhou 310058, China; ‡ School of Biological Sciences, University of Edinburgh, Edinburgh EH9 3FF, United Kingdom; § Research Center for Biological Computation, Zhejiang Laboratory, Hangzhou 311100, China

**Keywords:** bacteriophage P2/P4, phage-based delivery vector, Cas9 antimicrobial, tail fiber engineering, Escherichia coli O157:H7, Shigella flexneri

## Abstract

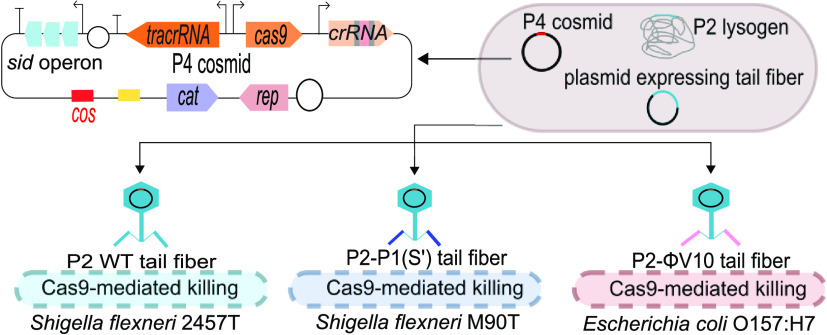

Bacteriophages can be reprogrammed to deliver antimicrobials for therapeutic and biocontrol purposes and are a promising alternative treatment to antimicrobial-resistant bacteria. Here, we developed a bacteriophage P4 cosmid system for the delivery of a Cas9 antimicrobial into clinically relevant human gut pathogens *Shigella flexneri* and *Escherichia coli* O157:H7. Our P4 cosmid design produces a high titer of cosmid-transducing units without contamination by a helper phage. Further, we demonstrate that genetic engineering of the phage tail fiber improves the transduction efficiency of cosmid DNA in *S. flexneri* M90T as well as allows recognition of a nonnative host, *E. coli* O157:H7. We show that the transducing units with the chimeric tails enhanced the overall Cas9-mediated killing of both pathogens. This study demonstrates the potential of our P4 *cas9* cosmid system as a DNA sequence-specific antimicrobial against clinically relevant gut pathogenic bacteria.

## Introduction

Antimicrobial resistance (AMR) is an important global health challenge, and alternatives to antibiotic treatments are urgently needed. Gut pathogenic bacteria that cause diarrhea diseases such as *Shigella* and *Escherichia coli* pose a great social and economic burden.^1^ In 2016, diarrhea diseases were responsible for 1.3 million deaths worldwide^2^ and were listed as the fifth leading cause of death in small children (age below 5 years) and eighth in all ages.^3^ Furthermore, the emergence of multidrug-resistant bacteria complicates the treatment of infection, and the limited availability of novel antimicrobial agents urges the need for alternative therapeutic options.

Bacteriophages are a promising alternative to antibiotic treatment due to several advantages such as self-amplification ability, biofilm degradation capability, and host specificity.^4,5^ However, they also have several limitations and disadvantages such as narrow host range, unwanted horizontal gene transfer, and resistance toward phage infection. Genetic engineering of bacteriophages could improve the efficiency of bacteriophage-based therapy and overcome these limitations.^6^ Bacteriophages have been repurposed as vectors to deliver antimicrobials into pathogenic bacteria.^7−11^ Examples of phage-based vectors are cosmids and phagemids, which contain bacteriophage-based elements, allowing their packaging into phage particles termed transducing units/particles.^10,12−14^ A helper phage is needed to provide genes encoding all of the factors required for the formation of phage progeny as the cosmid/phagemid does not encode structural proteins for phage particles formation. Antimicrobial-encoding gene(s) can be integrated into the cosmid/phagemid design, which will then be transduced into susceptible bacterial host strains. Since cosmids/phagemids cannot produce phage progeny without their helper phage, the potential risk of horizontal gene transfer after transduction is reduced compared to the use of a replicative phage in clinical settings^15^ and biocontrol. One such antimicrobial is the clustered, regularly interspaced, short palindromic repeats (CRISPR)-associated protein 9 (CRISPR-Cas9) system, which permits DNA sequence-specific bacterial killing.^7−9,11^ Cas9 is the main nuclease from type II CRISPR-Cas system, a bacterial immune system against invading foreign DNA.^16,17^ The Cas9 nuclease recognizes and cleaves a specific DNA sequence. This sequence is defined by the spacer sequence of its CRISPR RNA (crRNA), which forms a complex with a trans-activating CRISPR RNA (tracrRNA). The sequence specificity of Cas9 nuclease can be reprogrammed by modifying the spacer sequence of its crRNA to target a defined DNA sequence. Therefore, the CRISPR-Cas9 system can be used as a sequence-specific antimicrobial, whereby the endonuclease is reprogrammed to target and produce a double-strand break in the bacterial chromosome, leading to cell death if not repaired.^7,8^

Although genetic engineering and synthetic biology allow the development of synthetic bacteriophage-based vectors,^18^ there are still several drawbacks that limit their uses in clinical settings. A major drawback of cosmid/phagemid systems is the potential contamination of the helper phage in the transducing units. Due to its replicative nature, helper phage infection could kill the targeted bacterial cells as well as mediate horizontal transfer of virulence and/or antibiotic-resistant genes between susceptible bacterial host(s). To eliminate the helper phage contamination, deletion of the genomic DNA packaging site has been attempted on M13,^7^ 80α,^19^ and P2.^14^ The deletion of the packaging site on phage genome will stop the packaging of the phage genome but not the cosmid/phagemid, as it eliminates the contamination of the helper phage and therefore allows the production of pure cosmid/phagemid transducing units.

**Figure 1:**
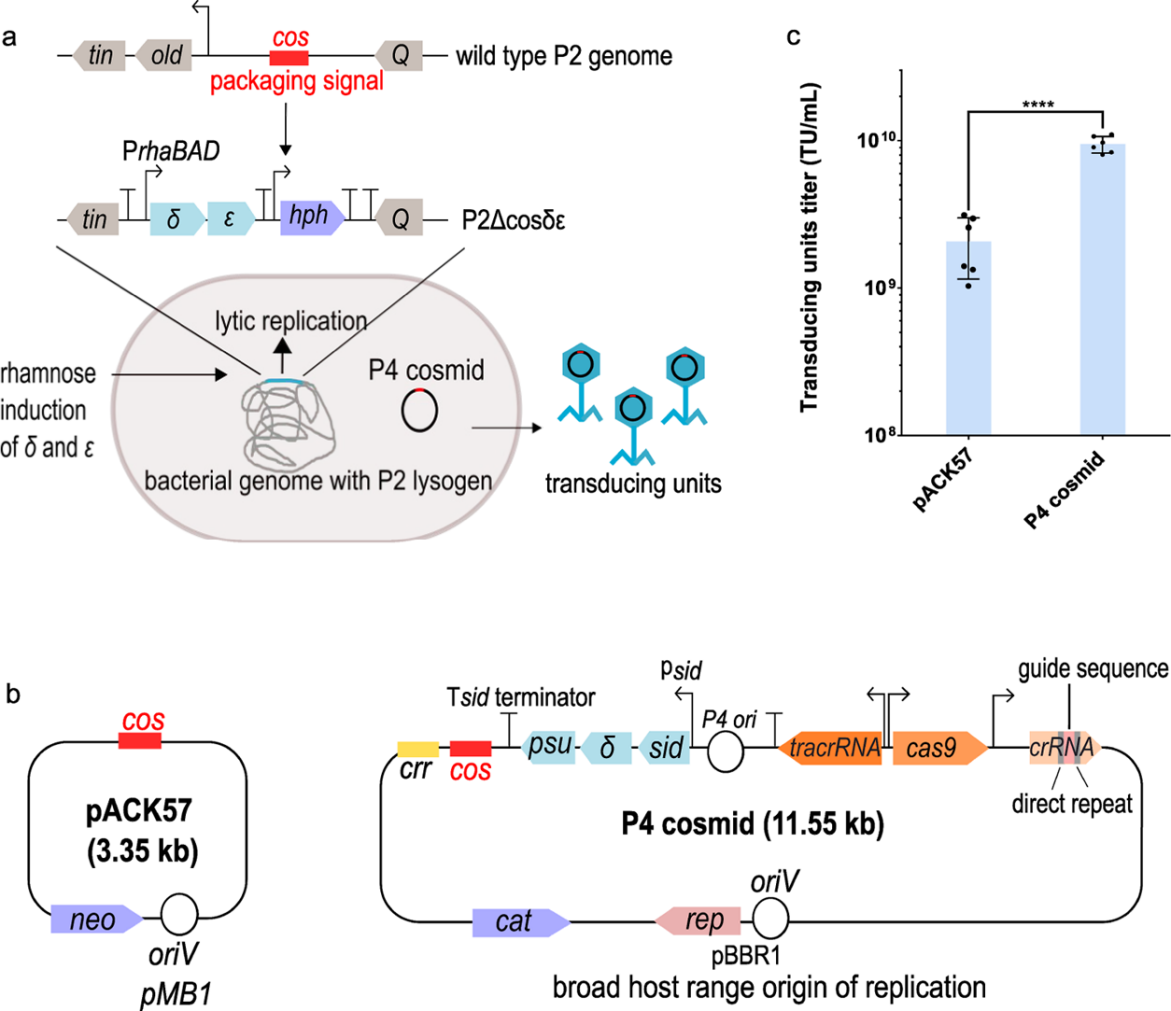
Comparisons between pACK57 and P4 cosmid design and the titer of transducing units. (a) Schematic diagram showing the production of cosmid-transducing units in a mutant P2Δ*cos* δε lysogenic strain. This mutant was generated by Tridgett et al.^14^ using *E. coli* C-5545 P2 lysogen lacking the *old* gene in the P2 genome. The absence of the *old* gene allows expression of lambda-red recombinase, which was used for genome modification of P2. The packaging (*cos*) site from the P2 genome is replaced by P4 δ and ε genes under an inducible rhamnose promoter. Rhamnose induction of δ and ε gene expression activates P2 lytic replication. Only the cosmid DNA is packaged into the capsid, producing pure cosmid-transducing units without helper phage contamination.^14^ (b) Schematic of pACK57 and P4 cosmids. The pACK57 cosmid contains a *cos* site for its packaging into P2 capsids, a kanamycin-resistant gene (*neo*), and the pMB1 origin of replication. The P4 cosmid contains an identical P4-derived *cos* site for its packaging into transducing units and P4 *sid* and *psu* genes for generating smaller-sized P4 capsids. The *crr* and a P4 origin of replication were included in the cosmid but were not active. A functional Cas9 system, consisting of genes encoding the Cas9 endonuclease, a tracrRNA, and a crRNA, was added to the P4 cosmid. The P4 cosmid has a chloramphenicol resistance gene (*cat*) as the selection marker and a pBBR1 origin of replication. (c) Comparison of transducing unit titer from lysates produced on *E. coli* P2Δ*cos* δε lysogen transformed with either a pACK57 or a P4 cosmid. A nonchromosomal-targeting *cas9* (*cas9*-NT) construct was used in this experiment, which contains a randomly generated spacer sequence of the crRNA guide. The data are represented as mean and the standard deviation as error bars. The mean of each cosmid was generated from two batches of lysate with three biological replicates (host cell). Each biological replicate had three technical replicates. The *p*-values were determined using an unpaired *t*-test calculated via GraphPad Prism with significance defined by *p* < 0.05. ****p ≤ 0.001.

In this study, we have developed a cosmid system derived from bacteriophage P4, which allows the production of high-titer transducing units encoding a CRISPR-Cas9 system targeting specific bacteria. The P4 cosmid system was adapted from a previous study^14^ that reprogrammed a P2 lysogen to generate pure cosmid-transducing units without the contamination of the P2 phage progeny. A mutant P2 lysogenic strain was generated by replacing the DNA packaging signal, *cos*, with δ- and ε-encoding genes of bacteriophage P4. Bacteriophage P4 is a satellite phage that relies on the P2-related helper phage for lytic replication.^20^ The P4 δ activates P2 late genes,^21^ while ε activates lytic replication of P2 prophage.^22^ The lack of a functional *cos* site, as well as an increase in the genomic size of the mutant P2 prophage (P2Δ*cos* δε) completely inhibited the formation of P2 phage progeny, ensuring the production of pure transducing units.^14^ The expression of δ and ε genes is controlled by an inducible rhamnose promoter, P_rhaB_, which allows the activation of P2 lytic replication and the formation of cosmid-transducing units upon rhamnose induction^14^^14^ (Figure 1a). The P2 pACK cosmid^14^ is a high copy number vector (pUC57) containing the DNA packaging signal *cos* site derived from bacteriophage P4 and a kanamycin resistance gene as the selection marker (Figure 1b). The pACK cosmid can be packaged into pure transducing units in the P2Δ*cos* δε lysogen without helper phage contamination. We have modified the size and design of the cosmid to include the P4-derived *sid* operon for optimum packaging of the vector into a P4 phage with a smaller capsid. Our data indicates a significant increase in the titer of transducing units when using the modified P4 cosmid design compared to that reported previously.^14^ We further demonstrate that genetic engineering of bacteriophage P2 tail fibers improves the transduction efficiency of cosmid DNA in *Shigella flexneri* M90T and *E. coli* O157:H7, thus producing a significant Cas9-mediated killing of *S. flexneri* (2457T and M90T) and *E. coli* O157:H7, which are clinically relevant human gut pathogens.

## Results and Discussion

### P4-Derived Cosmid System Generated Phage Lysates with a High Titer of Phagemid Transducing Units

We first improved the P2 cosmid system developed by Jaramillo and colleagues^14^ for delivering a Cas9 antimicrobial system into pathogenic bacterial strains, such as *S. flexneri* and *E. coli* O157:H7. Due to the nonreplicative nature of a cosmid when compared to a wild-type (WT) phage, an excess amount of transducing units is required to maximize the transduction efficiency. Therefore, the low titer of P2 transducing units (∼10^7^ transducing units/mL lysate) might be insufficient to produce a significant Cas9-mediated killing of a bacterial population. We hypothesized that the low titer of P2 transducing units might be due to a suboptimal packaging efficiency of the cosmid DNA into the P2 capsid. A specific size of the DNA substrate is required for its efficient packaging into P2 or the smaller-sized P4 capsid, whereby a cosmid that matches the genomic size of either P2 or P4 phage provides the maximum DNA packaging efficiency.^23,24^ While the use of a 33 kb cosmid DNA that matches P2 genomic size might be unnecessary for delivering a ∼4.5 kb CRISPR-Cas9 construct, the packaging of a ∼11.6 kb vector into the smaller-sized P4 phage could potentially yield a high titer of transducing units. We therefore designed a cosmid system derived from bacteriophage P4. Bacteriophage P4 is a satellite phage, which relies entirely on P2-derived structural proteins for the assembly of functional phages.^25^ While the capsids of both P2 and P4 consist of the P2-derived GpN protein, P4 produces a smaller capsid (45 nm diameter) that favors packaging of its smaller 11.6 kb genome, as opposed to the P2 capsid of 60 nm diameter.^26^ P4-derived Sid (size determination) protein binds to GpN, which promotes the assembly of smaller capsid size,^27−29^ while the other P4-derived protein, Psu, is important for the stability of the P4 capsid.^30^ Hence, we hypothesized that the inclusion of a *sid* operon (consisting of *psu*, δ, and *sid*) in the cosmid design would induce the formation of the P4 phage during P2 lytic lifecycle and therefore allow the packaging of a 11.6 kb cosmid DNA into smaller P4 capsids (Figure 1b). To maintain the P4 cosmid in different bacterial species, a broad host-range pBBR1 origin of replication was used in the cosmid design. A complete CRIPSR-Cas9 system consisting of genes encoding a Cas9 endonuclease, tracrRNA, and crRNA was included in the cosmid for targeting the chromosomal sequence of the bacterial species of interest^31^ (Figure 1b).

To investigate if the newly designed P4 cosmid improved the yield of transducing units, comparisons were made between the titer of the P4 and the pACK57 transducing units. *E. coli* C-5545 Δ*cos* δε P2 lysogen was transformed with either the P4 or pACK57 cosmid and the transformants were used for lysates preparation. The titers of transducing units were assessed on *E. coli* K-12 (EMG2) as the indicator strain, which were represented by the number of cosmid transductants recovered. Our P4 cosmid lysates yielded an average titer of 9.45 × 10^9^ transducing units per mL of lysate (TU/mL), which was 4.6-fold higher than the average titer of 2.07 × 10^9^ TU/mL recorded for the pACK57 cosmid lysates (Figure 1c) (*p* ≤ 0.001). A higher titer of cosmid-transducing units indicates that the packaging of the 11.55 kb cosmid DNA into P4 phages might be more efficient than to the production of pACK57 cosmid-transducing units. It is noteworthy that the titer of pACK57 transducing units in our hands was ∼100-fold higher when compared to that reported by the study from Tridgett et al.^14^ The difference in the pACK57 transducing unit titer might be due to the variations in the protocols used for lysate preparation and the quantification of cosmid transductants.

Taken together, the use of a P4 cosmid of 11.55 kb improved the titer of the transducing units, which indicates a higher packaging efficiency of the modified cosmid DNA into P4 capsids, when compared to the packaging of a pACK57 cosmid into P2 capsids. Now that we have a cosmid that can be packaged efficiently, we next investigated if this system can be used to deliver Cas9 antimicrobial into pathogenic bacteria *S. flexneri* and kill it in a sequence-specific manner.

### Cas9-Mediated Killing of *S. flexneri* 2a 2457T and 5a M90T Using the P4-Based Cosmid

**Figure 2:**
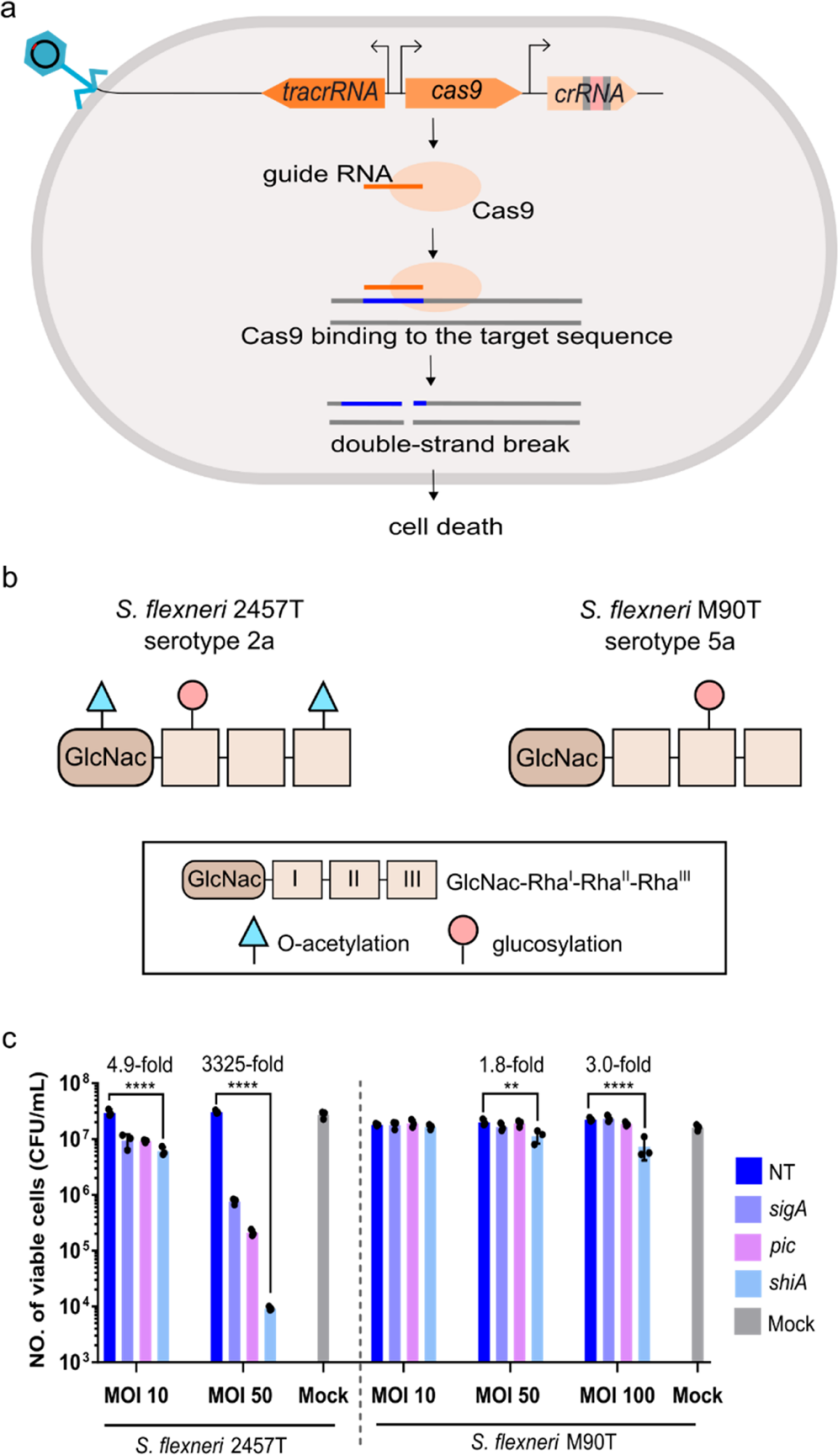
Cas9-mediated killing of the P4 cosmid in *S. flexneri* 2457T and *S. flexneri* M90T. (a) cas9 nuclease can be programmed to target a specific chromosomal DNA sequence through modification of the crRNA coding sequence. The Cas9 nuclease performs a double-strand break in the chromosome, which will lead to cell death if not repaired. (b) Schematic representation of O-antigen modification of *S. flexneri* 2457T serotype 2a and *S. flexneri* M90T serotype 5a. Both of the serotypes have the same O-antigen backbone comprising of one *N*-acetylglucosamine (GlcNAc) and three l-rhamnose residues (Rha^I^–Rha^II^–Rha^III^). The two serotypes differ from each other by the *O*-acetyl and glucosyl group. (c) Cas9-mediated killing of P4 cosmids in *S. flexneri* 2457T and M90T. The P4 cosmids were designed to have their spacer sequences complementary to *sigA*, *pic*, and *shiA* genes of *S. flexneri*. The data were presented as colony forming units (CFU) recovered after treatments with transducing units at different multiplicities of infection (MOIs). The data are represented as mean and the standard deviation as error bars. The experiment was carried out with three biological replicates, each having three technical replicates. The *p*-values were determined using one-way analysis of variance (ANOVA) with the Dunnett’s post hoc test calculated via GraphPad Prism with significance defined by *p* < 0.05. **p ≤ 0.01, ***p ≤ 0.001, ****p ≤ 0.001. NT, nontargeting (the crRNA guide does not recognize any sequences in the bacterial chromosome). Mock, cell treated with SM buffer supplemented with 20 mM CaCl_2_ instead of phage lysate (negative control).

To demonstrate that P4 transducing units can deliver a Cas9 antimicrobial system into pathogenic bacteria, we first assessed the Cas9-mediated antimicrobial effect of P4 cosmid on *S. flexneri*. *S. flexneri* is one of the causative agents of Shigellosis in which infection is associated with a high mortality and morbidity rate, especially in pediatric patients of low-income countries.^32,33^ Clinical isolates of *S. flexneri* are increasingly resistant toward antibiotics recommended for treatment of Shigellosis, such as azithromycin and ciprofloxacin.^34,35^ Moreover, the development of a broadly protective vaccine against Shigellosis is hampered by the genotypic and phenotypic variations of *Shigella* spp., most notably *S. flexneri* that consists of 14 known serotypes.^36,37^ We optimized the Cas9-mediated antimicrobial effect of the P4 cosmid on *S. flexneri* by designing several crRNA coding sequences that will allow the Cas9 endonuclease to target conserved chromosomal genes of these bacteria (Figure 2). Hence, we have designed three spacer sequences that are complementary to the conserved *sigA*, *pic*, and *shiA* virulence factor-encoding genes of *S. flexneri*.^38−41^ The targets we selected for this study are specific to *S. flexneri* and are not expected to be present in other gut commensal bacteria. P4 *cas9* cosmids with or without a *S. flexneri*-targeting crRNA coding sequence were used for phage lysates preparation, and the Cas9-mediated antimicrobial effect was first assessed in *S. flexneri* 2a 2457T by measuring the cell survival after transduction (Figure 2c). The reduction in the number of *S. flexneri* colony forming units (CFU) recovered between the chromosomal-targeting and nontargeting phagemid lysates treatment represents the Cas9-mediated antimicrobial effect on the bacteria. The titer of the transducing units was measured using *E. coli* EMG2 as an indicator strain and was normalized to provide a multiplicity of infection (MOI) of 10 and 50.

Transduction of *S. flexneri* with the *shiA*-targeting cosmid (*cas9-shiA*) at a MOI of 10 reduced the number of *S. flexneri* CFU by ∼5-fold when compared to that recovered after treatment with the nontargeting (*cas9*-NT) cosmid (Figure 2c). At a higher MOI of 50, *cas9-sigA*, *cas9-pic*, and *cas9-shiA* cosmids reduced the number of *S. flexneri* CFU by ∼40, ∼147, and ∼3325-fold, respectively, when compared to treatment with the nontargeting *cas9*-NT cosmid lysates. There was no significant difference in the number of CFU recovered between *cas9*-NT cosmid lysates treatment and mock infections (SM buffer supplemented with 20 mM CaCl_2_, no transducing units), which demonstrates that the antimicrobial effect on *S. flexneri* was specific toward the presence of the chromosomal-targeting crRNA sequences. *cas9-shiA* was shown to be most efficient in killing of *S. flexneri* 2457T. The difference in the Cas9-mediated killing efficiency between *sigA*, *pic*, and *shiA*-targeting cosmids could be due to multiple factors such as the target site accessibility and mismatch between the target and the guide sequence, and the flanking sequences around the target site can influence the Cas9 efficiency.^42−44^

Next, we wanted to investigate if the Cas9-mediated killing effect observed in *S. flexneri* 2457T can be reproduced in another serotype of *S. flexneri*. Transduction of the chromosomal-targeting and nontargeting *cas9* cosmids was carried out at MOIs of 10, 50, and 100, on *S. flexneri* 5a M90T, which is a laboratory strain widely used for the pathogenicity study^45^ (Figure 2c). *S. flexneri* M90T (serotype 5a) was selected because it has a different O-antigen modification compared to *S. flexneri* 2457T (serotype 2a)^46^ (Figure 2b), which will be informative to determine if our P4 cas9-transducing units can be used to target wider O-antigen structures in the context of *S. flexneri*. Treatment of *S. flexneri* M90T with either the *cas9-sigA* or *cas9-pic* cosmid lysates at all MOI yielded a similar number of CFU when compared to *cas9*-NT cosmid lysates. The similarity in CFU recovered when compared to the nontargeting cosmid treatment indicates that both the *cas9-sigA* and *cas9-pic* cosmids did not produce any significant Cas9-mediated killing in *S. flexneri* M90T. Similarly, the *cas9-shiA* cosmid lysates reduced the number of M90T CFU slightly by ∼2- and ∼3-fold at MOIs of 50 and 100, respectively, when compared to that recovered after a *cas9*-NT cosmid lysates treatment. In comparison with the antimicrobial effect produced on 2457T, chromosomal-targeting *cas9* cosmids produced a negligible killing effect in *S. flexneri* M90T.

Taken together, chromosomal-targeting P4 *cas9* cosmids produced a great antimicrobial effect on *S. flexneri* 2457T but not on *S. flexneri* M90T. The efficiency of phage infection depends on the interaction between the phage tail fiber/tail spike and its receptor on the bacterial cell surface.^47^*S. flexneri* serotype 2a and 5a share a common *O*-polysaccharide backbone but with different glucosyl and *O*-acetyl groups^46,48^ (Figure 2b). The difference in the cell surface component such as O-antigen and core lipopolysaccharides (LPS) can influence phage infectivity and host range.^49−53^ Therefore, we hypothesized that the difference in the O-antigen modification between the two *S. flexneri* strains could potentially affect transduction of *cas9* cosmids, which would contribute to the difference in the Cas9-mediated killing. Tail fiber/tail spike engineering can expand or alter the host range of the bacteriophage.^54−56^ Notably, a chimeric P2 tail fiber with the host-range-determining region (HRDR) of bacteriophage S16 tail fiber allowed P2 infection of *Salmonellaenterica* serovar Typhimurium via recognition of S16 native receptor, OmpC. We hypothesized that the same approach via engineering of the tail fiber of P2 could increase the transduction efficiency into *S. flexneri* M90T.

### Chimeric Tail P2-P1(S′) Improves the Transduction Efficiency and Cas9-Mediated Killing of P4 Cosmids in *S. flexneri* M90T

Genetic engineering of phage tail fibers, such as the use of chimeric phage tail fibers with alternative HRDR, has been reported to alter the host range of a phage.^54,55,57−59^ Hence, to improve the transduction efficiency of our P4 cosmid into *S. flexneri* M90T, we changed the HRDR of P2/P4 phage tail fibers, which is located at its C-terminus region with the HRDR of a *S. flexneri*-infecting phage. One such candidate is bacteriophage P1, which was shown to transduce phagemid DNA into *S. flexneri* strains at a high efficiency.^12,49^ P1 expresses two different tail fibers, namely, the S and S′, which allow host tropism switching.^60−62^ Our previous study demonstrates that the P1 with S′ tail fiber could transduce *cas9* constructs into *S. flexneri* serotypes 2a and 5a at a high efficiency.^49^ Therefore, the S′ tail fiber was a promising candidate for constructing a chimeric P2 tail fiber.

**Figure 3:**
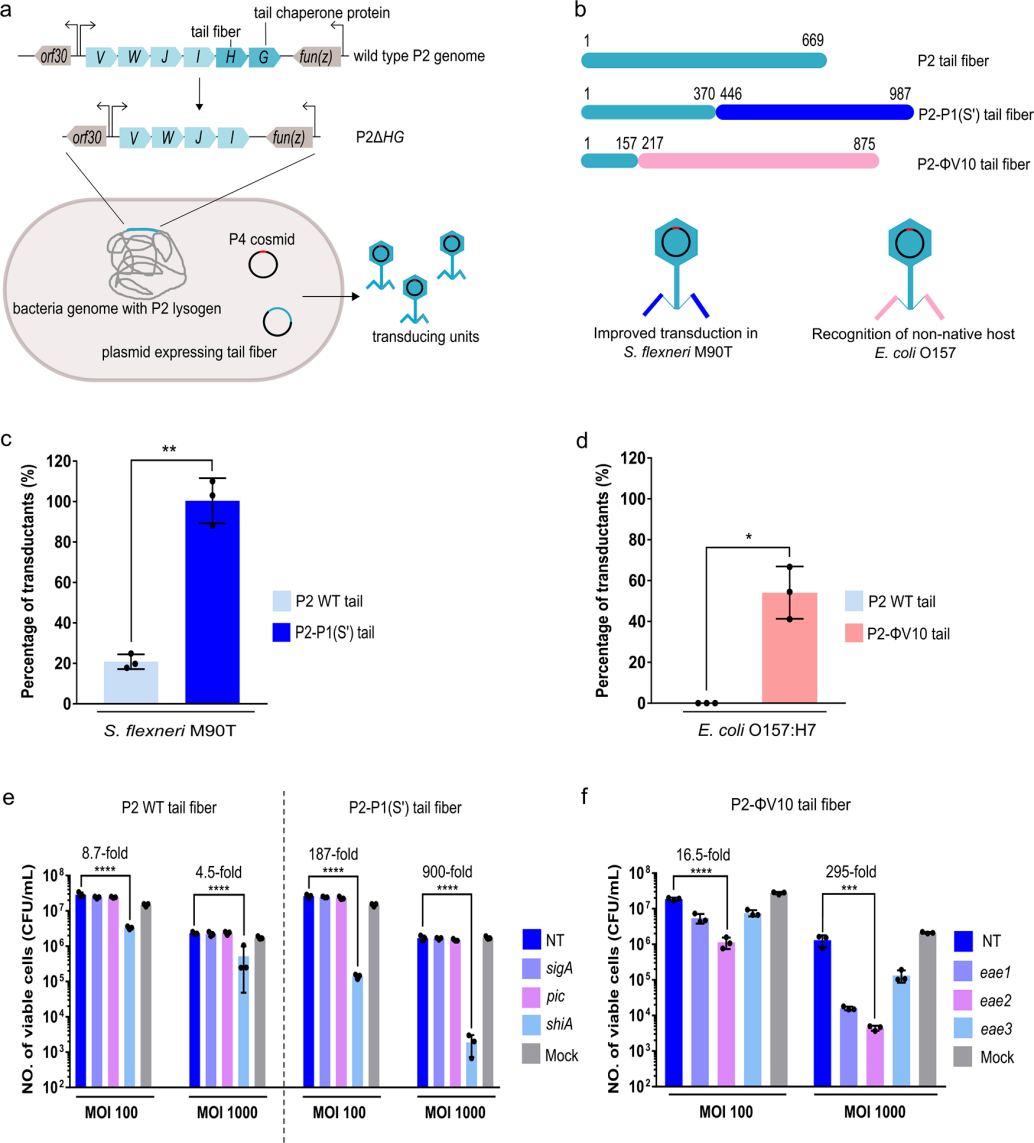
Transducing units with engineered tail fibers improve the delivery of the antimicrobial *cas9* construct in *S. flexneri* M90T and *E. coli* O157:H7. (a) Tail fiber (*H*) and its chaperone protein (*G*) were deleted from the P2 helper phage genome. The mutation could be complemented in trans by providing a plasmid encoding a chimeric tail fiber and a corresponding chaperone protein, which would allow the formation of infectious P4 transducing units. (b) Schematic drawing of the chimeric P2-P1(S′) tail and P2-ϕV10 tail. The horizontal bars are used to represent the tail fibers. The DNA sequences encoding both chimeric tail fibers are provided in Table S6. (c) Percentage of transductants (%) in *S. flexneri* M90T of P4 phage particles with wild-type (WT) P2 tail fibers or chimeric P2-P1(S′) tail fibers (MOI of 1). (d) Percentage of transductants (%) in *E. coli* O157:H7 of P4 phage particles with WT P2 tail fibers or chimeric P2-ϕV10 tail fibers (MOI of 1). (e) Cas9-mediated killing of *S. flexneri* M90T after infection of phage particles with P2 WT or P2-P1(S′) tail fibers (f) Cas9-mediated killing in *E. coli* O157:H7 after infection of phage particles with P2 WT or P2-ϕV10 tail fibers. The data are presented as colony forming units (CFU) recovered after treatments with transducing units at different multiplicities of infection (MOI). The data is represented as mean and the standard deviation as error bars. The experiment was carried out with three biological replicates, each having three technical replicates. The *p*-values were determined using unpaired *t*-test (c, d) or one-way ANOVA with Dunnett’s post hoc test (e, f) calculated via GraphPad Prism with significance defined by *p* < 0.05. **p ≤ 0.01, ***p ≤ 0.001, ****p ≤ 0.0001. NT, nontargeting (the crRNA does not recognize any sequence in the bacterial chromosome). Mock, cell treated with SM buffer supplemented with 20 mM CaCl_2_ instead of the phage lysate (negative control).

We have generated a Δ*HG* mutant strain of P2 lysogen, whereby the tail fiber-encoding gene *H* and its chaperone protein-encoding gene *G* were deleted (Figure 3a). The expression of chimeric P2 tail fibers in trans complemented the Δ*HG* mutation. These chimeric tail fibers may allow the formation of infectious P4 phages with an altered host range. To determine an optimal fusion site for the P2-P1(S′) chimeric tail fibers, the DNA sequence encoding the N-terminal region of the P2 tail fiber was fused to the DNA sequence encoding the C-terminus region of the P1(S) tail fiber at different nucleotide positions (Table S1). P1(S) and P1(S′) tail fiber share an identical amino acid sequence from 1 to 654, which encompasses the place of fusion. Therefore, optimization was carried out with P1(S) and the most efficient fusion site was selected for the generation of chimeric P2-P1(S′). This was the fusion of the 370th and 446th amino acid residues of P2 and P1(S)/P1(S′) tail fibers, respectively (Table S1). The P1 chaperone protein-encoding gene, *U* or *U*′, was also included on the plasmid as the protein might be essential for the correct folding of the chimeric tail fiber having the HRDR of P1(S) or P1(S′) tail fiber, respectively.^57^ The Δ*HG* mutant P2 lysogen was cotransformed with *cas9*-NT cosmid as well as either the chimeric P2-P1(S′) tail fiber-encoding plasmid (with *U*′) or a P2 tail fiber-encoding plasmid (with *G*). Cosmid lysates were prepared from the transformants and assessed for the titer of transducing units on *S. flexneri* M90T by calculating the number of transductants recovered. The horizontal bars in Figure 3b are used to represent the P2-P1(S′) tail fiber constructed using 1–370 amino acid residues from the P2 tail fiber and 446–987 amino acid residues from the P1(S′) tail fiber.

To determine if the transducing units with the chimeric P2-P1(S′) tail fibers could improve transduction of the P4 cosmid in *S. flexneri* M90T, we measured the transduction efficiency of the WT or P2-P1(S′) transducing units. The titers of WT or P2-P1(S′) transducing units were normalized to provide a MOI of 1 using the transduction assay with *S. flexneri* M90T as an indicator strain. At a MOI of 1, the transduction efficiency of P4 phage transducing units with the chimeric P2-P1(S′) tail fibers was ∼100%, which was ∼4–5-fold higher than the transduction efficiency of P4 phages with P2 WT tail fibers (Figure 3c). Transduction efficiency of transducing units with the chimeric P2-P1(S′) or WT tail fibers at a MOI of 10 was reported in Figure S1. We then investigated whether a higher transduction efficiency of cosmids mediated by P4 transducing units with chimeric P2-P1(S′) tail fibers (termed P2-P1(S′) transducing units hereafter) would increase the Cas9-mediated killing of *S. flexneri* M90T. Therefore, we compared the Cas9-mediated killing of *S. flexneri* M90T between infections with WT or P2-P1(S′) transducing units (Figure 3e). The *cas9*-NT as well as chromosomal-targeting *cas9* cosmids were packaged into transducing units with WT P2 or P2-P1(S′) tail fibers. The P2-P1(S′) transducing units, however, were unable to infect *E. coli* K-12 (EMG2), which prompted us to estimate the titer of WT as well as P2-P1(S′) cosmid-transducing units via quantitative polymerase chain reaction (qPCR) (Figure S2). The titers of WT or P2-P1(S′) transducing units were normalized to provide MOIs of 100 and 1000.

Transduction of either the *cas9-sigA* or *cas9-pic* using either WT or P2-P1(S′) transducing units did not reduce the number of M90T CFU significantly when compared to transduction of the *cas9*-NT cosmid at MOIs of 100 and 1000. At a MOI of 100, however, the transduction of a *cas9-shiA* cosmid using transducing units with P2 WT tail fibers reduced the number of *S. flexneri* CFU by ∼8.7-fold when compared to the transduction of a *cas9*-NT cosmid (Figure 3e). Cas9-mediated killings of *cas9-shiA* in *S. flexneri* M90T at a MOI of 100 from Figures 2c and 3e were 3- and 8.7-fold, respectively. The difference in this Cas9-mediated killing effect cannot be directly compared with each other as the method for MOI normalization was not the same. In Figure 2c, the transducing units were measured using a transduction assay with *E. coli* EMG2 as an indicator strain, while the transducing units in Figure 3e were measured using qPCR. It is noteworthy to highlight that qPCR measured the number of packaged cosmids in the capsid, while the transduction assay measured the number of functional transducing units.

The delivery of the *cas9-shiA* cosmid with chimeric P2-P1(S′) transducing units produced a ∼21-fold higher level of Cas9-mediated killing when compared to transducing units with the WT P2 tail at the same MOI of 100 (Figure 3e). Furthermore, a 10-fold increase in the MOI of P2-P1(S′) *cas9-shiA* transducing units increased the Cas9-killing effect to 900-fold reduction in CFU. In contrast, the use of WT *cas9-shiA* transducing units at a MOI of 1000 did not significantly improve the Cas9-mediated killing when compared to the infection at a lower MOI of 100. The discrepancies in the Cas9-mediated killing produced between WT and P2-P1(S′) transducing units suggest a nonlinear relationship between transduction efficiency and the MOI. While a higher MOI increases the rate of phage–bacteria interactions, the rate of successful phage infection might be determined by other factors, such as the affinity of phage adsorption. Therefore, a higher titer of transducing units might not compensate for a lower binding affinity of the WT P2 tail fiber to *S. flexneri* M90T when compared to that of a chimeric P2-P1(S′) tail fiber.

A future experiment comparing the efficiency of the transducing units with antibiotics could be carried out to investigate if the transducing units generated in this study can be used as an antibiotic alternative. Azithromycin or ciprofloxacin, which are recommended antibiotics for shigellosis,^34,35^ could be compared to the use of transducing units for *S. flexneri* treatment. Moreover, the synergistic interaction between the transducing units generated in this study and the antibiotics could also be investigated. Furthermore, bacterial cells are often more resistant to antibiotics during the stationary phase compared to the exponential phase.^63^ It would be interesting to investigate if the transducing units can be used to efficiently kill cells in the stationary phase as well.

Taken together, delivery of a *shiA*-targeting *cas9* cosmid with the P2-P1(S′) transducing units into *S. flexneri* M90T produced a significantly higher Cas9 antimicrobial killing than when using transducing units with WT tail fibers. As the tail fiber engineering of bacteriophage P2 resulted in higher transduction efficiency of *S. flexneri* M90T, we then wanted to investigate if the same approach can be used to retarget P2 to a nonnative host.

### Chimeric tail P2-ϕV10 Allows Transduction of the P4 Cosmid into a Nonnative Host, *E. coli* O157:H7, Resulting in High Cas9-Mediated Killing

One of the nonnative host of P2 that we wanted to target is enterohemorrhagic *E. coli* (EHEC) O157:H7. EHEC infection caused the highest mortality rate among cases of AMR bacterial infections in 2019.^1^ This pathogen causes mild to bloody diarrhea, hemorrhagic colitis (HC), and hemolytic-uremic syndrome (HUS).^64^ A major pathogenesis of EHEC infection is the production of the shiga toxin,^65,66^ which is cytotoxic to human endothelial cells and renal glomerular and tubular epithelial cells.^67,68^ Bacteriophage ϕV10 belongs to the Podoviridae family and it can infect *E. coli* O157:H7.^69^ Previous investigations^70−72^ demonstrated that a chimeric P2 tail fiber with the C-terminus region of bacteriophage ϕV10 tail spike protein allowed transduction of *E. coli* O157:H7. Therefore, we hypothesized that a P2-ϕV10 tail spike protein would allow transduction of the *cas9* cosmid into *E. coli* O157:H7. We first assembled a gene sequence encoding the chimeric P2-ϕV10 tail spike protein into a plasmid (Figure 3b). Phage lysates were prepared from the Δ*HG* mutant of *E. coli* C-5545 Δ*cos* δε P2 lysogen that was cotransformed with a *cas9*-NT cosmid as well as a P2-ϕV10 tail spike-encoding or a WT P2 tail fiber-encoding plasmid. We compared the transduction efficiency between transducing units with either WT P2 or P2-ϕV10 tail fibers (termed P2-ϕV10 transducing units hereafter) by determining the relative abundance of cosmid transductants recovered after infection at a MOI of 1 (Figure 3d). While the WT transducing units did not produce any cosmid transductant, P2-ϕV10 transducing units yielded a transduction efficiency of ∼54%, thus indicating that the chimeric P2-ϕV10 tail spike allowed transduction of the *cas9* cosmid in a nonnative host.

Next, we investigated if P4 transducing units with chimeric P2-ϕV10 tail fibers could deliver a chromosomal-targeting *cas9* cosmid into *E. coli* O157:H7. Since the *E. coli* O157:H7 strain used in this study lacks the shiga toxin genes, *stx1* and *stx2*, three crRNA sequences were designed to target the *eae* gene, which is another marker gene specific to *E. coli* O157:H7.^73,74^ The *eae*-targeting *cas9* cosmids (*cas9-eae*) as well as the nontargeting *cas9*-NT cosmids were packaged into transducing units with P2-ϕV10 chimeric tail fibers in the Δ*HG* mutant strain of the *E. coli* P2 lysogen. The titer of transducing units *eae*-targeting or nontargeting *cas9* cosmids were normalized to provide a MOI of 100 or 1000 on *E. coli* O157:H7 using qPCR. Transduction of *cas9-eae2* cosmid produced the highest level of Cas9-mediated antimicrobial effect, which caused 16.5- and 295-fold reduction in *E. coli* O157:H7 CFU at MOIs of 100 and 1000, respectively, when compared to transduction of the *cas9*-NT cosmid (Figure 3f). The Cas9-mediated killing of our P4 cosmid at a MOI of 100 was comparable to that reported by the study of Citorik et al.^7^ whereby a bacteriophage M13-based system was used to deliver *eae*-targeting *cas9* phagemids into *E. coli* O157:H7. It is noteworthy to highlight that the crRNAs used in the study by Citorik et al.^7^ and in this study are different. The authors demonstrated that the *eae-*targeting M13 reduced the number of bacterial CFU by 20-fold when cells were recovered using nonselective media, which was increased by an additional 100-fold under kanamycin selection for the transductants.^7^ On the other hand, our P4 cosmid produced a 295-fold reduction in CFU at a MOI of 1000 under nonselective conditions.

The use of antibiotics for *E. coli* O157:H7 treatment is not recommended as this was shown to be associated with a higher risk of HUS due to the increase of shiga toxin production or shiga toxin release.^75^ Transducing units generated in this study could potentially be used for *E. coli* O157:H7 treatment as the Cas9-mediated killing is likely to be a result of DNA digestion by RecBCD complex or other nucleases in the cell,^76^ which is not expected to lead to cell lysis and subsequent unwanted shiga toxin release. However, this was not determined in this study. A further investigation of whether the CRISPR-Cas9 system used in this study leads to cell lysis of *E. coli* O157:H7 is an important experiment to lay the foundation for the use of Cas9 antimicrobial for *E. coli* O157:H7 treatment.

The cells that survived the Cas9-mediated killing from the efficient crRNA-targeting sequences (*shiA* for *S. flexneri* and *eae2* for *E. coli* O157:H7) can be subjected to DNA sequencing to fully understand the underlying mechanism that the cells were used for escaping the activity of chromosomal-targeting Cas9 cosmid. These “escape” mutants could be the result of a mutation in the CRISPR-Cas9 targeted sequence or a mutation at the host recognition site. Alternatively, these cells might not have been transduced, have lost the phagemid, or have been transduced with a defective phagemid.^7^

Although Cunliffe et al.^56^ generated a chimeric P2-S16 tail fiber that allowed P2 infection of *S.* Typhimurium, the tail fiber-encoding gene, *H*, of the resident P2 prophage was not deleted. This resulted in the production of WT P2 phage progeny with heterogeneous tail fibers. In contrast, we adapted the Δ*HG* mutant that will generate homogeneous phage progeny that only packages the cosmid and one tail fiber type. We believe that our system can be used with the chimeric tail generated by Cunliffe et al.^56^ for retargeting of P2 toward *S.* Typhimurium. The combination of both approaches has the potential to retarget phage P2 toward various nonnative hosts, extending the application of this system to more pathogenic bacteria.

## Conclusions

Genetic engineering of bacteriophages provides a powerful platform to generate synthetic bacteriophages with desirable characteristics to overcome the limitations of bacteriophage therapy while reducing potential risks associated with phage-mediated horizontal gene transfer. Overall, our data demonstrate the potential use of genetically engineered P4 phages in delivering chromosomal-targeting Cas9 constructs into two important gut pathogens, *S. flexneri* and *E. coli* O157:H7. Specifically, genetic engineering of P2/P4 phage tail fibers further improved the transduction efficiency of a *cas9* cosmid into *S. flexneri* M90T, as well as allowed transduction of the *cas9* antimicrobial system into a nonnative host, *E. coli* O157:H7. In addition to expanding the host range of a bacteriophage, the tail engineering approach could potentially be expanded to overcome the issue of phage resistance caused by mutation(s) in bacterial gene(s) encoding for phage receptors. A further experiment performing bacterial resistance induction could be carried out to investigate if the bacterial cells that are resistant to transduction with WT P2 transducing units can still be targeted using P2-P1(S′) transducing units and vice versa. A chimeric phage tail fiber and/or tail spike might maintain the delivery of a Cas9 antimicrobial system into phage-resistant strain(s) of the bacteria. This further exemplifies the potential of our P4 *cas9* cosmid system as a nonreplicative DNA sequence-specific antimicrobial for clinical and biocontrol purposes.

## Materials and Methods

### Bacterial Strains, Primers, Plasmids, Buffer, and Growth Medium

Bacterial strains used in this study are listed in Table S2. Bacteria were cultured in Luria-Bertani (LB) medium (1% tryptone, 0.5% yeast extract, 1% NaCl). SM buffer (100 mM NaCl, 8 mM MgSO_4_, 50 mM Tris pH 7.5) was used for phage lysate dilution and transduction assays. Primers and plasmids are listed in Tables S3 and S4, respectively.

### Construction of P4 Cosmids and Cloning of crRNA-Encoding Sequences

The P4 cosmids were constructed using Gibson assembly and the primers listed in Table S3. The P4 cosmid original to this study will be made available via the Addgene plasmid repository. Molecular cloning of spacer sequences was based on a protocol established by Jiang et al.^31^ The crRNA guide sequence of our *cas9* construct contains two *Bsa*I recognition motifs, which allow digestion of the cosmid backbone and annealing of the spacer sequence. A pair of oligonucleotides that contain the spacer sequence was designed to have 5′ overhangs, which allowed annealing of the oligonucleotide pair into the CRISPR array. The chromosomal-targeting spacer sequences were designed using CHOPCHOP.^44^ The primer pairs were phosphorylated by incubating 1 μL of each primer (100 μM) with 5 μL of 10× T4 ligase buffer (New England Biolabs), 1 μL of T4 polynucleotide kinase (PNK) (New England Biolabs), and 42 μL of water at 37 °C for 30 min, followed by heat inactivation at 65 °C for 20 min. The primers were then annealed by incubating at 95 °C for 5 min and cooled to room temperature slowly in the heat block for a minimum of 30 min. The P4 cosmid DNA was digested with the *Bsa*I restriction enzyme (New England Biolabs) according to the manufacturer’s instruction and gel purified. The annealed primers were ligated to the digested P4 cosmid by incubating 50 μg of cosmid with 2 μL of annealed primers (diluted to 50–100 μg/μL), 2 μL of 10× T4 ligase buffer (New England Biolabs), 1 μL of T4 ligase (New England Biolabs), and water added to the final volume of 20 μL. The reaction was incubated at room temperature for 2 h and heat-inactivated at 65 °C for 20 min. Two microliters of the reaction was used for transformation for plasmid propagation. The DNA sequence of P4 cosmid is available in Table S6.

### Lambda-Red Recombineering for Tail Fiber (*H*) and Chaperone (*G*) Knock-Out

Genetic modification of the P2 genome was carried out using the lambda-red recombineering technique, as described previously^77^ with slight modification. Briefly, the PCR product for gene knock-out was designed with a 50 bp homology region flanking both sides of the genes to be deleted. Both *H* and *G* genes were replaced with a kanamycin resistance gene (*neo*) flanked with two flanking FRT sites (FPL recognition target). *E. coli* C-5545 P2 Δ*cos* δε lysogen strain was first transformed with pKD46, which harbors the lambda-red recombinase (γ, β, *exo*)^77^ (Table S4). An overnight culture of the transformed cells was diluted 1:100 in fresh LB and grew until OD_600_ reached 0.35–0.4 at 30 °C. The pKD46 plasmid has a temperature-sensitive replicon, hence, transformants were grown (or propagated) at 30 °C. l-Arabinose was added to the culture at a final concentration of 0.65 M to induce the expression of the lambda-red recombinase and the culture was allowed to grow further at 30 °C for 15–30 min. The cells were chilled on ice for 40 min and were made electrocompetent before being transformed with the knock-out template (the template sequence is available in Table S5). Kanamycin-resistant colonies were selected for PCR to verify for correct insertion of the *neo* cassette, followed by Sanger sequencing of the PCR product to verify gene deletion. To remove the kanamycin resistance gene from the genome, cells were transformed with pCP20 (Table S4) and grown at 30 °C overnight. Colony PCR reactions were carried out with primers JF218 and JF219 to verify the excision of the *neo* cassette. The cells were then grown at 43 °C nonselectively overnight to cure the pCP20 plasmid. The cells were then tested for ampicillin and kanamycin sensitivity to select for colonies that have lost the *neo* cassette and the pCP20 (ampicillin-resistant) plasmid.

### Construction of Plasmids Encoding Tail Fiber

The plasmid encoding P2 WT tail fibers *H* and its chaperone protein *G* was constructed using Golden Gate assembly (Figure S3). Primers JF561 and JF562 (Table S3) were used to amplify *H* and *G* from the P2 genome. Primers JF563 and JF564 (Table S3) were used to amplify the 4A3 vector backbone with pSC101 origin of replication and ampicillin resistance gene. Oligo JF565 and JF566 encoding the P_V_ promoter sequence were phosphorylated and annealed as described above. All fragments were assembled via Golden Gate assembly using SapI enzyme (New England Biolabs). The reaction was set up as the following: 2 μL of 10× T4 ligase buffer (New England Biolabs), 1 μL of PNK (New England Biolabs), 1 μL of SapI (New England Biolabs), DNA fragments at a ratio of 3:3:1 (insert/insert/vector), and water to a final volume of 20 μL. The reaction was incubated for 3 min at 37 °C and 4 min at 16 °C for 26 cycles, followed by incubation at 37 °C for 15 min and 65 °C for 20 min. The plasmids encoding the chimeric tail fibers P2-P1(S′) and P2-ϕV10 were constructed using Gibson assembly and primers listed in Table S3. The DNA sequences of the plasmids encoding tail fibers and their chaperone protein are available in Table S6. The plasmids expressing tail fibers constructed in this study will be made available via the Addgene plasmid repository.

### Phage Lysate Preparation

The *E. coli* C-5545 P2 lysogen Δ*cos* δε was transformed with the P4 cosmid. The *E. coli* C-5545 P2 lysogen Δ*cos* δε Δ*HG* mutant strain was cotransformed with the P4 cosmid and a plasmid encoding WT or the chimeric P2 tail fibers. Next, 100 μL of an overnight culture of the transformed cells was diluted in 10 mL of fresh LB with the appropriate antibiotics. The cells were allowed to grow for 2–2.5 h at 37 °C, followed by centrifugation at 4500*g* for 5 min to pellet the cells. The cells were washed twice in LB to remove residual antibiotics, followed by resuspension of the cell pellet in 2.5 mL of LB supplemented with 10 mM sodium citrate and 0.2% l-rhamnose. l-Rhamnose was used for induction of the lytic replication. The culture was incubated at 37 °C for 4 h until the completion of cell lysis, which was represented by the clearing of bacterial cultures and the accumulation of cell debris. Chloroform was added to lysates at a final concentration of 2.5%, followed by a brief vortexing and shaking of the culture at 37 °C for 10 min to ensure bacterial sterilization of samples. The lysate was centrifuged at 5000*g* for 5 min to remove cell debris, filter-sterilized using a 0.22 μM syringe filter, and stored at 4 °C.

### Quantification of Transducing Units

Transducing units were quantified according to a previous protocols^12,13^ with slight modification. Briefly, the bacterial indicator strain was grown overnight at 37 °C. The overnight culture was diluted 1:100 in fresh LB and grown at 37 °C with shaking, until an OD_600_ of 0.5–0.6. The cells were centrifuged at 4500*g* for 5 min and concentrated 20-fold in SM buffer supplemented with 20 mM CaCl_2_. Then, 100 μL of the concentrated bacteria were mixed with 100 μL of the lysate (diluted 10-fold in SM buffer + 20 mM CaCl_2_) and adsorption was allowed to occur for 30 min at 37 °C with shaking. Next, 800 μL of SOC with 10 mM sodium citrate was added and the mix was incubated for 1 h at 37 °C to allow expression of the antibiotic resistance gene. Sodium citrate, which sequesters free calcium ions, was added to quench further phage infection. The recovered cells were diluted 10-fold and spotted on nonselective LB plates, as well as LB plates supplemented with chloramphenicol (25 μg/mL) to select for cosmid transductants. The plates were incubated for a minimum of 16 h at 37 °C and the colonies were counted.

### DNase I Treatment of Lysates

Lysates were pretreated with Amplification Grade DNAse I (Sigma-Aldrich, AMPD1) to remove noncapsulated DNA, according to the manufacturer’s protocol with slight modification. Briefly, 1 μL of the lysate was mixed with 1 μL of DNAse I, 1 μL of 10× reaction buffer, and 7 μL of nuclease-free water. The reaction was incubated at room temperature for 15 min before 1 μL of stop solution was added. The reaction was incubated at 70 °C for 10 min to inactivate DNAse I. Nuclease-free water was added to the reaction to give a final dilution of 1:100 for the lysate. The reaction was stored at 4 °C.

### Quantitative Polymerase Chain Reaction (qPCR)

The presence of a chromosomal-targeting crRNA leads to Cas9-targeting and killing of targeted bacteria. Therefore, cosmids encoding crRNA are not stably maintained in targeted bacteria after transduction. Hence, the titers of chromosomal-targeting, *cas9*-transducing units, were routinely assessed on *E. coli* K-12 strain EMG2, which is not targeted by the crRNA used in this study. However, P4 transducing units with chimeric P2-P1(S′) tail fibers and P2-ϕV10 tail spikes can only infect *S. flexneri* and *E. coli* O157, respectively. In these bacteria, the chromosomal-targeting *cas9* cosmid is inherently unstable after transduction. Hence, the titers of chromosomal-targeting *cas9*-transducing units with chimeric tail fibers were quantified by measuring the copy number of cosmid DNA that were packaged into phage units by qPCR.^78,79^ A LightCycler 96 (Roche) was used to perform qPCR with SYBR Green PCR Master Mix (Thermo Fisher Scientific, 4309155). The sequences of the primers used for qPCR (qPCR1 and qPCR2) are provided in Table S3. They bind and amplify the *rep* gene of the pBBR1 origin of replication. The *rep* gene is specific to the cosmid DNA and not present in the genome of the bacterial strains used in this study. The 10 μL reactions were prepared with 5 μL of SYBR Green PCR Master Mix, 1 μL of DNAse I treated lysate, 0.3 μL (300 nM) of each primer, and 3.4 μL of nuclease-free water. PCR cycling conditions were 95 °C for 10 min; 45 cycles of 95 °C for 20 s, 60 °C for 20 s, 72 °C for 20 s; and melting at 95 °C for 10 s, 65 °C for 60 s, 97 °C for 1 s. The nontargeting Cas9 P4 cosmid DNA was used to construct a standard curve for quantification of the transducing units in the lysate. The concentration of P4 cosmid DNA was measured using Qubit dsDNA HS assay kit (Thermo Fisher Scientific) and was serially diluted 10-fold from 8.02 × 10^8^ to 8.02 × 10^2^ copy number/μL to construct the standard curve. The lysate produced using pACK57 cosmid, lacking the target sequence, was used as a negative control. The results were analyzed using a LightCycler 96 SW 1.1. See Figure S2 for the standard curve and the titer of the transducing units (copy number/mL).

### Quantification of Cosmid Transduction Efficiency and Cas9-Mediated Antimicrobial Killing

The targeted bacterial cells were grown overnight in LB at 37 °C. The overnight culture was diluted 1:100 in fresh LB and grown under agitation at 37 °C until an OD_600 nm_ of 0.4–0.5. The cells were then diluted in SM buffer supplemented with 20 mM CaCl_2_ to an OD_600 nm_ of 0.1 (which gives ∼1 × 10^8^ CFU /mL). The phage lysate was diluted to a specific concentration, giving the desired MOI in SM buffer supplemented with 20 mM CaCl_2_ (e.g., for a MOI of 10, the phage lysate was diluted to 1 × 10^8^ TU/mL when used with 1 × 10^7^ CFU /mL of cell). Then, 100 μL of the lysate was mixed with 100 μL of cells and incubated at 37 °C for 30 min. Next, 1 × 10^7^ CFU/mL was used for all of the experiments except for the Cas9-killing experiment at a MOI of 1000 for P4 cosmid-transducing units with WT and chimeric P2-P1(S′) or P2-ϕV10 tail fibers that used 1 × 10^6^ CFU /mL. SM buffer supplemented with 20 mM CaCl_2_ (with no phage) was used as a mock reaction. Then, 800 μL of SOC supplemented with 10 mM of sodium citrate was added to the mixture to recover the cells and to quench further phage infection. The cells were incubated for 1 h under agitation at 37 °C. The cells were then diluted 10-fold and spotted on nonselective LB agar and LB agar supplemented with chloramphenicol. The cells were allowed to grow at 37 °C for a minimum of 16 h, and the number of CFU recovered were counted the next day. The transduction efficiency was defined by the number of chloramphenicol colonies per number of total colonies recovered on nonselective agar. Cas9-mediated killing was defined by the reduction in CFU recovered after treatment with chromosomal-targeting transducing units compared to nontargeting transducing units.

## Supporting Information Available

qPCR standard curve; number of packaged P4 cosmids in each lysate calculated by qPCR; different fusion sites for the generation of P2-P1(S) tail fibers; relevant genotypes of bacteria and bacteriophages used and their source; plasmids and cosmids used in this study; primers used in this study; sequence for the *HG* knock-out; and DNA sequences for all plasmids and cosmids used in this study (PDF)
