# Supplementary material for: Tail-Engineered Phage P2 Enables Delivery of Antimicrobials into Multiple Gut Pathogens
Source: ACS Synth Biol. 2023 Feb 2;12(2):596–607. doi: 10.1021/acssynbio.2c00615 (PMC9942202; doi:10.1021/acssynbio.2c00615)
Supplement: Supplementary file 1 — sb2c00615_si_001.pdf [file sb2c00615_si_001.pdf]

**Supplementary Information for**

# "Tail-engineered phage P2 enables delivery of antimicrobials into multiple gut pathogens"

Jidapha Fa-arun[2], Yang Wei Huan[2], Elise Darmon[2] and Baojun Wang[1,2,3]*

[1]College of Chemical and Biological Engineering & ZJU-Hangzhou Global Scientific and Technological Innovation Center, Zhejiang University, Hangzhou 310058, China

[2]School of Biological Sciences, University of Edinburgh, Edinburgh EH9 3FF, United Kingdom

[3]Research Center for Biological Computation, Zhejiang Laboratory, Hangzhou 311100, China

*correspondence (baojun.wang@zju.edu.cn)

## Table of Contents

Supplementary Figures S1-S3

Supplementary Tables S1-S6

Supplementary References 1-5

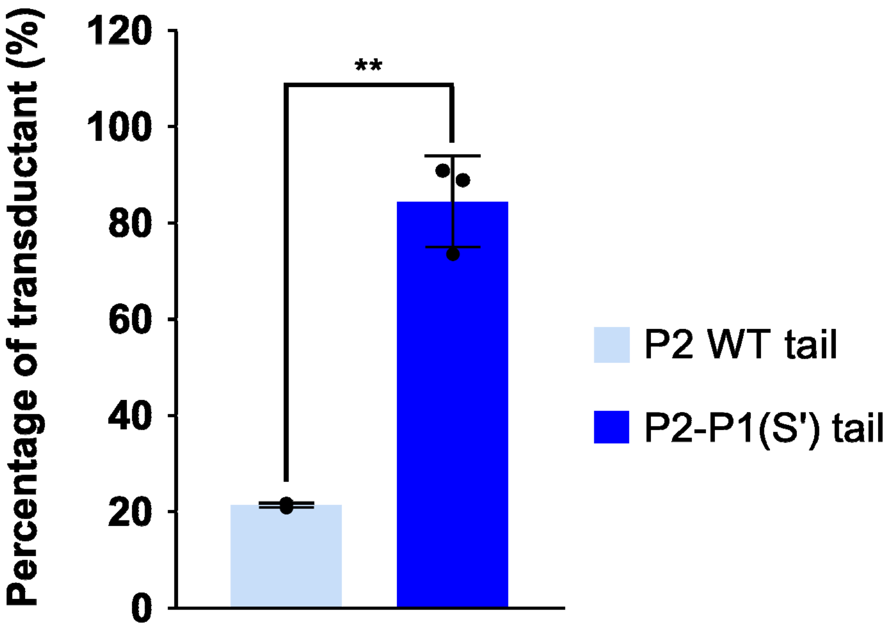


**Supplementary Figure S1**. **Percentage of transductants (%) of *S. flexneri* M90T cells after treatment with P4 transducing particles harbouring wild-type (WT) P2 tail fibers or chimeric P2-P1(S') tail fiber at MOI of 10.** The transducing units concentration used was $1\times10^7$ TU/mL and the cell concentration used was $1\times10^6$ CFU/mL. Lower cell concentration was used here compared to the transduction assay carried out at MOI of 1 ($1\times10^7$ CFU/mL, **Figure 3c**) because the transducing unit titer of the chimeric tail P2-P1(S') was ~$2\times10^7$ TU/mL when titer on *S. flexneri* M90T. Therefore in order to achieve an MOI of 10, lower cell concentration have to be used. The *p*-values were determined using unpaired *t*-test calculated using GraphPad Prism with significance defined by $p < 0.05$. **$p \leq 0.01$.

a

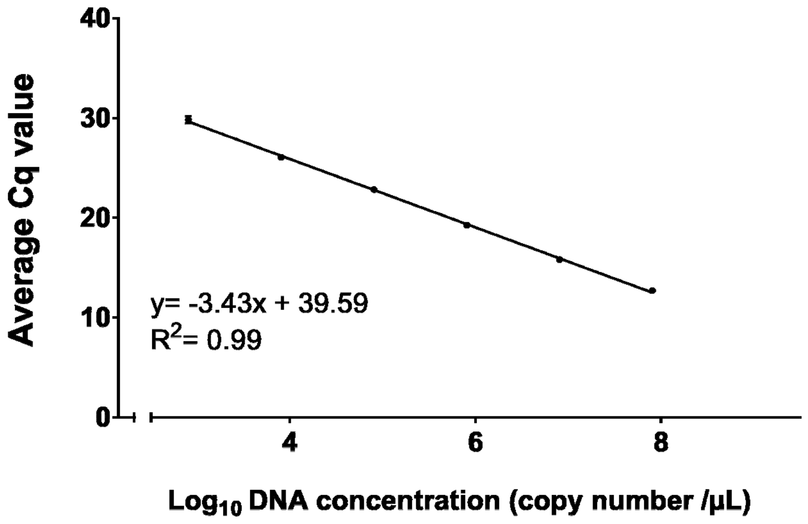


b

| Lysate | Number of packaged cosmids (copy number/ mL) |
|---|---|
| *cas9*-NT with P2 WT tail fiber | $1.88 \times 10^{10}$ |
| *cas9-sigA* with P2 WT tail fiber | $2.82 \times 10^{10}$ |
| *cas9-pic* with P2 WT tail fiber | $2.58 \times 10^{10}$ |
| *cas9-shiA* with P2 WT tail fiber | $2.35 \times 10^{10}$ |
| *cas9*-NT *cas9* with P2-P1S' tail fiber | $2.58 \times 10^{10}$ |
| *cas9-sigA* with P2-P1S' tail fiber | $1.99 \times 10^{10}$ |
| *cas9-pic* with P2-P1S' tail fiber | $2.20 \times 10^{10}$ |
| *cas9-shiA* with P2-P1S' tail fiber | $2.77 \times 10^{10}$ |
| *cas9*-NT *cas9* with P2-ϕV10 tail fiber | $4.25 \times 10^{10}$ |
| *cas9-eae1* with P2-ϕV10 tail fiber | $3.63 \times 10^{10}$ |
| *cas9-eae2* with P2-ϕV10 tail fiber | $2.98 \times 10^{10}$ |
| *cas9-eae3* with P2-ϕV10 tail fiber | $4.12 \times 10^{10}$ |

**Supplementary Figure S2**. **qPCR standard curve and transducing unit titer.** (**a**) Standard curve for phage titer quantification, generated using varying concentrations of the non-targeting P4 *cas9*-NT cosmid as a template. The DNA concentration (in $\log_{10}$ copy number/µL) was determined by the average quantification cycle (Cq) values calculated by qPCR. The template was diluted from $8.02 \times 10^2$ to $8.02 \times 10^8$ copy number/µL. Cq values are shown with standard deviation of the mean as error bars. Each data has 3 technical replicates. The equation and correlation coefficient of the graph is calculated using Graphpad Prism. (**b**) Concentration of packaged P4 cosmids in different lysates (copy number/mL) measured by qPCR.

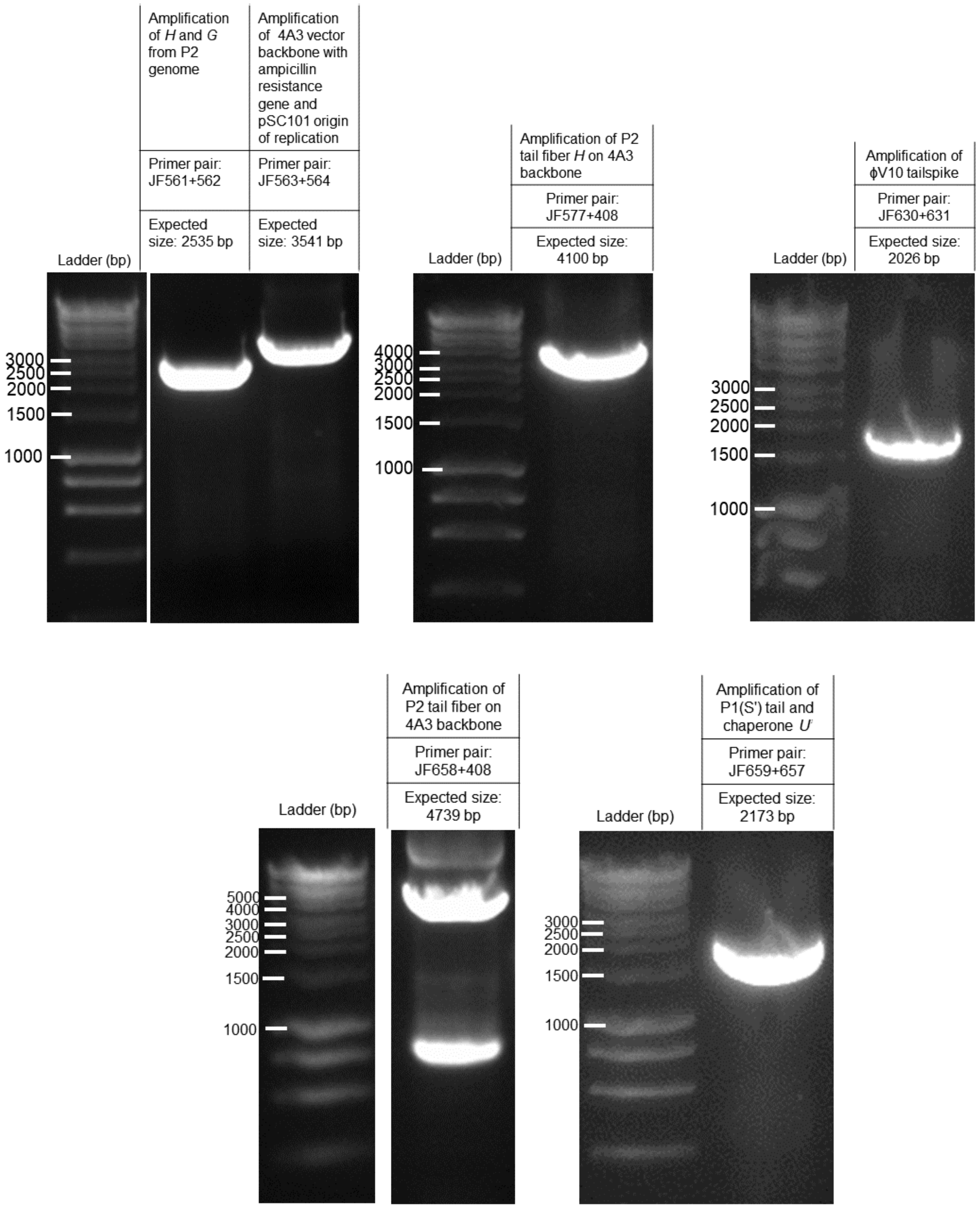


**Supplementary Figure S3**. **Agarose gel images of PCRs for construction of plasmids for tail fiber expression.** The primer pair used and the expected band size for each PCR reaction is indicated. The PCR bands of the expected size were gel extracted and the final plasmids were sequenced to confirm the right assembly.

**Supplementary Table S1**. **Fusions between a P2 tail fiber and a P1(S) tail fiber at different locations.** Different versions of chimeric P2-P1(S) were constructed and tested for the ability to transduce the cosmid DNA into *E. coli* EMG2. Fusion of the P2 tail fibers from 1-370 amino acid residues with the 446-987 amino acid residues of P1(S) generated the highest titer of cosmid transducing units. The fusion site was selected for the construction of a P2-P1(S') tail fiber (**Figure 3b**). The horizontal bars in **Figure 3b** are used to represent the P2-P1(S’) tail fiber constructed using 1-370 amino acid residues from P2 tail fiber and 446-987 amino acid residues from P1(S’) tail fiber of Supplementary Table S1. P1(S) and P1(S') have identical amino acid sequence from 1-654, therefore the fusion site of P2-P1(S) can be used for P2- P1(S').

| **N-terminal P2 tail fiber** | **C-terminal P1(S) tail fiber** | **Activity / transducing units titer (TU/ mL)** | |
|---|---|---|---|
| 1-157 | 157-987 | - | 0 |
| 1-157 | 169-987 | - | 0 |
| 1-157 | 175-987 | - | 0 |
| 1-157 | 181-987 | + | $1.2 \times 10^4$ |
| 1-162 | 157-987 | - | 0 |
| 1-162 | 169-987 | - | 0 |
| 1-162 | 175-987 | + | $4 \times 10^3$ |
| 1-162 | 181-987 | + | $4 \times 10^3$ |
| 1-170 | 169-987 | - | 0 |
| 1-170 | 157-987 | - | 0 |
| 1-170 | 175-987 | - | 0 |
| 1-170 | 181-987 | - | 0 |
| 1-214 | 214-987 | - | 0 |
| 1-354 | 430-987 | + | $6.5 \times 10^5$ |
| 1-370 | 446-987 | + | $2 \times 10^7$ |

**Supplementary Table S2**. Bacterial strains used in this study.

| Strain | Relevant genotype | Source |
|---|---|---|
| *Escherichia coli* EMG2 | Wild type *E. coli* | Kindly gifted by Dr. Alfonso Jaramillo, the University of Warwick[1] |
| *Escherichia coli* C-5545 Δ*cosδε* | Deficient in the packaging *cos* site and encoding rhamnose-inducible P4 *δ* and *ε* genes. | Kindly gifted by Dr. Alfonso Jaramillo, the University of Warwick[1] |
| *Escherichia coli* C-5545 Δ*cosδε* Δ*HG* | As above with tail fiber (*H*) and its chaperone (*G*) deleted | This study |
| *Shigella flexneri* 2a 2457T ATCC 700930 | Wild type *S. flexneri*, serotype 2a | American Type Culture Collection (ATCC)[2] |
| *Shigella flexneri* 5a M90T | Wild type *S. flexneri*, serotype 5a | Kindly gifted by Dr. Serge Mostowy, the London School of Hygiene & Tropical Medicine[3] |
| *Escherichia coli* O157:H7 NCTC12900 | Lacks shiga toxin gene *stx1* and *stx2* | Kindly gifted by Prof. David Gally, the University of Edinburgh[4] |

**Supplementary Table S3**. Primers used in this study.

| Name | Sequence (5' to 3') | Description | Usage |
|---|---|---|---|
| eae1-F | aaactggggtaacggactttacggg | Guide sequence targeting of *eae* gene of *E. coli* O157:H7 | Cloning into P4 cosmid for chromosomal targeting of *E. coli* O157:H7 |
| eae1-R | aaaacccgtaaagtccgttacccca | Guide sequence targeting of *eae* gene of *E. coli* O157:H7 | Cloning into P4 cosmid for chromosomal targeting of *E. coli* O157:H7 |
| eae2-F | aaactaccattacttataccgcgag | Guide sequence targeting of *eae* gene of *E. coli* O157:H7 | Cloning into P4 cosmid for chromosomal targeting of *E. coli* O157:H7 |
| eae2-R | aaaactcgcggtataagtaatggta | Guide sequence targeting of *eae* gene of *E. coli* O157:H7 | Cloning into P4 cosmid for chromosomal targeting of *E. coli* O157:H7 |
| eae3-F | aaacgcgcctaatgccggatatgag | Guide sequence targeting of *eae* gene of *E. coli* O157:H7 | Cloning into P4 cosmid for chromosomal targeting of *E. coli* O157:H7 |
| eae3-R | aaaactcatatccggcattaggcgc | Guide sequence targeting of *eae* gene of *E. coli* O157:H7 | Cloning into P4 cosmid for chromosomal targeting of *E. coli* O157:H7 |
| HYWBW002 | cttcagtcacctcctagctgactcaaatc | Amplification of *cas9* fragment (reverse) | Construction of P4 cosmid |
| HYWBW004 | gagtcagctaggaggtgactgaag | Amplification of crRNA, pBBR1 *ori*, *cat* fragment (forward) | Construction of P4 cosmid |
| HYWBW255 | acccagctttcttgtacaaagtggttgatccggatctttgatcgg | Amplification of crRNA, pBBR1 *ori*, *cat* fragment (reverse) | Construction of P4 cosmid |
| HYWBW633 | ccagaaaagaggcctcccgaaaggggggcctttttcgttttggtccgtcaagttgtcataattggtaacgaatcag | Amplification of *cas9* fragment (forward) | Construction of P4 cosmid |

| | | | |
|---|---|---|---|
| HYWBW635 | ccctttcgggaggcctcttttctggaatttggtaccgagctgattctgtggataaccgta | Amplification of pACK cosmid backbone, non-coding sequence (reverse) | Construction of P4 cosmid |
| HYWBW637 | accactttgtacaagaaagctgggtccgaaggtgaacaatccactg | Amplification of *crr* DNA sequence (forward) | Construction of P4 cosmid |
| HYWBW638 | aggtgcatggctttgcatgcgttttc | Amplification of P4 *cos* site (reverse) | Construction of P4 cosmid |
| HYWBW639 | gaaaacgcatgcaaagccatgcacct | Amplification of *sid* operon (reverse) | Construction of P4 cosmid |
| HYWBW640 | tgacgcgccaaaaaatttttttcgaaagaactgttcac | Amplifcation of *crr* DNA sequence (reverse) | Construction of P4 cosmid |
| HYWBW641 | gaaaaaaaatttttggcgcgtcagcgggtgttggcgggtgtcg | Amplification of P4 *cos* site (forward) | Construction of P4 cosmid |
| HYWBW644 | agcgctttattttgtgaatattttc | Amplification of *sid* operon (forward) | Construction of P4 cosmid |
| HYWBW645 | gaaaatattcacaaaataaagcgctcagcacaggcagcacatcat | Amplification of pACK cosmid backbone, non-coding sequence (forward) | Construction of P4 cosmid |
| JF218 | ctgtcaaacatgagaattaa | Binds to PCR template for *HG* knock-out to verify the genotype | Colony PCR for *HG* knock-out |
| JF219 | gtgtaggctggagctgcttc | Binds to PCR template for *HG* knock-out to verify the genotype | Colony PCR for *HG* knock-out |
| JF408 | ggatccgaattcgagctccgtcgacaag | Amplification of 4A3 backbone | Construction of plasmid encoding P2- P1(S') and P2-ϕV10 tail fiber tail fiber |
| JF561 | cgactcagctcttcaattcagaaccgttatcaccactgccggtgcagc | Amplification of *H* and *G* from P2 genome | Construction of plasmid encoding P2 WT tail fiber |
| JF562 | cgactcagctcttcatccttattgcggcatttccggccattcaggatttgcag | Amplification of *H* and *G* from P2 genome | Construction of plasmid encoding P2 WT tail fiber |
| JF563 | cgactcagctcttcaagcggccgcgaattccagaaatcatc | Amplification of 4A3 vector backbone with ampicillin resistance | Construction of plasmid encoding P2 WT tail fiber |

| | | | |
|---|---|---|---|
| | | gene and pSC101 origin of replication | |
| JF564 | cgactcagctcttcaggatccgaattcgagctccgtcgacaag | Amplification of 4A3 vector backbone with ampicillin resistance gene and pSC101 origin of replication | Construction of plasmid encoding P2 WT tail fiber |
| JF565 | gcttctagagtacgctctccttgaatgttgtctggtagttctacaaatgaatccagatagcataacttttatatattgtgcaatctcacatgcatgagcataaa | Oligo encoding the P2 late promoter sequence with SapI cut site for Golden Gate assembly | Construction of plasmid encoding P2 WT tail fiber |
| JF566 | aattttatgctcatgcatgtgagattgcacaatatataaaagttatgctatctggattcatttgtagaactaccagacaacattcaaggagagcgtactctaga | Oligo encoding the P2 late promoter sequence with SapI cut site for Golden Gate assembly | Construction of plasmid encoding P2 WT tail fiber |
| JF577 | aattttgtcatcaacgtaatcctgcgtcgccatc | Amplification of P2 tail fiber on 4A3 backbone (use with JF408) to make P2-ϕV10 | Construction of plasmid encoding P2-ϕV10 tail fiber |
| JF616 | ctgatggatgagactgtcacggccattgaacg | Primer to remove BsaI cut site from P4 *sid* gene | Construction of P4 cosmid |
| JF617 | gtgacagtctcatccatcaggcgggcgttctcaag | Primer to remove BsaI cut site from P4 *sid* gene | Construction of P4 cosmid |
| JF630 | caggattacgttgatgacaaaattttagcggcaaatgatggatttaagtttattggtcagtgccctgatatattgacac | Amplification of ϕV10 tailspike | Construction of plasmid encoding P2-ϕV10 tail fiber |
| JF631 | gacggagctcgaattcggatccttaaatatttccgttcagcgtgcggaatgagtattgtttatccaggtctactggggtaaataatc | Amplification of ϕV10 tailspike | Construction of plasmid encoding P2-ϕV10 tail fiber |
| JF657 | gacggagctcgaattcggatcc | Amplification of P1(S') tail | Construction of plasmid encoding P2- P1(S') tail fiber |
| JF658 | cgatttagccagaatatcccgcccgac | Amplification of P2 tail fiber on 4A3 backbone (use with JF408) to make P2-P1(S') | Construction of plasmid encoding P2- P1(S') tail fiber |

| | | | |
|---|---|---|---|
| JF659 | cgggatattctggctaaatcgagcattcttgctgttatccaataccttg | Amplification of P1(S') tail and chaperone *U'* | Construction of plasmid encoding P2- P1(S') tail fiber |
| pic-F | aaacgcttcagcattgtttgagtcg | Guide sequence targeting of *pic* gene of *S. flexneri* | Cloning into P4 cosmid for chromosomal targeting of *S. flexneri* |
| pic-R | aaaacgactcaaacaatgctgaagc | Guide sequence targeting of *pic* gene of *S. flexneri* | Cloning into P4 cosmid for chromosomal targeting of *S. flexneri* |
| qPCR1 | tggtcagccagaagacactt | Amplify *rep* gene on P4 cosmid | qPCR |
| qPCR2 | gcttcacgacggagatcca | Amplify *rep* gene on P4 cosmid | qPCR |
| shiA-F | aaacgcatgacttctccggctctcg | Guide sequence targeting of *shiA* gene of *S. flexneri* | Cloning into P4 cosmid for chromosomal targeting of *S. flexneri* |
| shiA-R | aaaacgagagccggagaagtcatgcg | Guide sequence targeting of *shiA* gene of *S. flexneri* | Cloning into P4 cosmid for chromosomal targeting of *S. flexneri* |
| sigA-F | aaacacgactttcccagtcggggctg | Guide sequence targeting of *sigA* gene of *S. flexneri* | Cloning into P4 cosmid for chromosomal targeting of *S. flexneri* |
| sigA-R | aaaacagcccgactgggaaagtcgt | Guide sequence targeting of *sigA* gene of *S. flexneri* | Cloning into P4 cosmid for chromosomal targeting of *S. flexneri* |

**Supplementary Table S4**. Plasmids and cosmids used in this study.

| Plasmid/cosmid | Description | Source |
|---|---|---|
| pACK57 | Plasmid with P4 *cos* site and kanamycin resistance gene on pUC57 backbone | Kindly gifted by Dr. Alfonso Jaramillo, University of Warwick[1] |
| P4 cosmid (Benchling link: https://benchling.com/s/seq-6yFP2RWWbSpLiQ5TUrlh?m=slm-wcMQes28bw1I34vy2zFb) | Plasmid with P4 *cos* site, *sid*, *δ* , *psu*, *crr* and P4 origin of replication. It also has *cas9*, tracrRNA and crRNA encoding sequence from *S. pyogenes* from p*cas9* (Addgene plasmid #42876) with pBBR1 origin of replication and a chloramphenicol resistance gene. | This study |
| P2 wild-type tail fiber expressing plasmid (pP2_tail_fiber) (Benchling link: https://benchling.com/s/seq-vNfYIKnwRP4OYpmSkkRo?m=slm-TrNcJk4I2QpwwQ3lBP3R) | Plasmid with a gene encoding P2 wild-type tail fiber *H* and its chaperone protein *G* under the P2 late promoter ($P_V$) controlling the *VWJIHG* operon. | This study |
| P2-P1S' expressing plasmid (pP2-P1(S')) (Benchling link: https://benchling.com/s/seq-FAG6ibePA6kzILw89oZQ?m=slm-sy1f4HfVnnlIMFe6hMJg) | Plasmid with a gene encoding the chimeric P2-P1(S') tail fibre as well as the P1 U' chaperone-encoding gene, whose expressions are controlled by P2 late promoter controlling *VWJIHG* operon. | This study |
| P2-ϕV10 tail fiber expressing plasmid (pP2-phiV10) (Benchling link: https://benchling.com/s/seq-ZFEaqfjblqOaGLFpmy7a?m=slm-taXpYJ5d2uDXPcT0XPWZ) | Plasmid with chimeric P2-ϕV10 tail under the P2 late promoter controlling *VWJIHG* operon. | This study |
| pKD46 | Plasmid expressing lambda-red recombineering proteins for P2 genome modification. It has ampicillin resistance gene and oriR101 temperature-sensitive origin of replication. | Coli Genetic Stock Center (CGSC)[5] |

| | | |
|---|---|---|
| pCP20 | Plasmid expressing FLP recombinase for removal of antibiotics resistance gene from P2 genome. It has ampicillin and chloramphenicol resistance gene and oriR101 temperature-sensitive origin of replication. | Coli Genetic Stock Center (CGSC)[5] |

**Supplementary Table S5**. Sequence of PCR template for *H* and *G* knock-out

ggcttacgacggcgatgtaattacggtttaccccggataagtgaggaatactgtcaaacatgagaattaattccggggatccgt
cgacctgcagttcgaagttcctattctctagaaagtataggaacttcagagcgcttttgaagctcacgctgccgcaagcactca
gggcgcaagggctgctaaaggaagcggaacacgtagaaagccagtccgcagaaacggtgctgaccccggatgaatgtc
agctactgggctatctggacaagggaaaacgcaagcgcaaagagaaagcaggtagcttgcagtgggcttacatggcgat
agctagactgggcggttttatggacagcaagcgaaccggaattgccagctggggcgccctctggtaaggttgggaagccct
gcaaagtaaactggatggctttcttgccgccaaggatctgatggcgcaggggatcaagatctgatcaagagacaggatgag
gatcgtttcgcatgattgaacaagatggattgcacgcaggttctccggccgcttgggtggagaggctattcggctatgactggg
cacaacagacaatcggctgctctgatgccgccgtgttccggctgtcagcgcaggggcgcccggttctttttgtcaagaccgac
ctgtccggtgccctgaatgaactgcaggacgaggcagcgcggctatcgtggctggccacgacgggcgttccttgcgcagct
gtgctcgacgttgtcactgaagcgggaagggactggctgctattgggcgaagtgccggggcaggatctcctgtcatctcacctt
gctcctgccgagaaagtatccatcatggctgatgcaatgcggcggctgcatacgcttgatccggctacctgcccattcgacca
ccaagcgaaacatcgcatcgagcgagcacgtactcggatggaagccggtcttgtcgatcaggatgatctggacgaagagc
atcaggggctcgcgccagccgaactgttcgccaggctcaaggcgcgcatgcccgacggcgaggatctcgtcgtgacccat
ggcgatgcctgcttgccgaatatcatggtggaaaatggccgcttttctggattcatcgactgtggccggctgggtgtggcggac
cgctatcaggacatagcgttggctacccgtgatattgctgaagagcttggcggcgaatgggctgaccgcttcctcgtgctttacg
gtatcgccgctcccgattcgcagcgcatcgccttctatcgccttcttgacgagttcttctaataactcggtaccaaattccagaaa
agaggcctcccgaaaggggggccttttttcgttttggtccggggatcttgaagttcctattccgaagttcctattctctagaaagtat
aggaacttcgaagcagctccagcctacacgttgtatgacctctgttgtgaacttacatatctatggcacagagtaaagc

The homology region to P2 genome is highlighted in red, the neomycin phosphotransferase (*neo*) is highlighted in blue. FLP recognision target (FRT) is underlined.

**Supplementary Table S6**. DNA sequences of genetic components and constructs.

| DNA sequence of the P4 cosmid |
|---|
| P4 origin of replication<br><br>agcgctttattttgtgaatattttcagcagacgcaacaggggggatttgttcaggctgtcttacaatggctgtgtgtttttgttcatctc<br>cacttaaagtcatttaaagccacttaaagcaatttgtaatttttatagtgaaatacaaatcgtttcttcttattcattcccggcgaatta<br>ataaaaacaaacagtagtaaacagcacaaaaagcccatcaacgggtgaacagtggtgaacagacggtgaacagtcatt<br>actgcgattgttcaccctttaacttactgtattacttatctttttttattaaggtgaacagaggtgaacagtaaaatataaaaaaacaa<br>acagtaagccggtttttcctgcgaccttttcctggcttgccggtctgaggatgagtctcctgtgtcagggctggcacatctgcaatg<br>cgtcgtgttgttgtccggtgtacgtcacaattttcttaacctgaagtgacgaggagccggaaa |
| *sid*<br><br>atgtctgaccacactatccctgaatatctgcaacccgcactggcacaactggaaaaggccagagccgcccatcttgagaac<br>gcccgcctgatggatgagactgtcacggccattgaacgggcagagcaggaaaaaaatgcgctggcgcaggccgacgga<br>aacgacgctgacgactggcgcacggcctttcgtgcagccggtggtgtcctgagcgacgagctgaaacagcgccacattga<br>gcgcgtggcacgccgggagctggtacaggaatatgacaatctggccgtggtgctgaatttcgaacgtgaacgcctgaaagg |

| |
|---|
| ggcgtgtgacagcacggccaccgcctaccggaaggcacatcatcaccttctgagtctgtatgcagagcatgagctggaaca<br>cgccctgaatgaaacctgtgaggcgcttgtccgggcaatgcatctgagcattctggtacaggaaaatccgctcgccaacacc<br>accggccatcagggctacgtcgcaccggaaaaggctgtcatgcagcaggtgaaatcatcgctggaacagaaaattaaac<br>agatgcaaatcagcctcaccggcgagccggttctccggctgaccggactgtcagcggcaacactcccgcacatggattatg<br>aggtggcaggcacaccggcacagcgcaaggtgtggcaggacaaaatagaccagcagggagcagagcttaaggccag<br>agggctgctgtcatga |
| *delta* (*δ*)<br><br>atgatttactgtccgtcgtgttgatgagttgctcacacccgtcgcgcacatttcatggacgatggcaccaagataatgattgcaca<br>gtgccggaatatttattgctctgcgacatttgaagcgagtgaaagctttttctctgacagtaaagattcaggaatggaatacatttc<br>aggcaaacagagataccgcgattcactgacgtcagcctcctgcggtatgaaacgcccgaaaagaatgcttgttaccggatat<br>tgttgtcggagatgtaaaggccttgcactgtcaagaacatcgcggcgtctgtctcaggaagtcaccgagcgtttttatgtgtgca<br>cggatccgggctgtggtctggtgtttaaaacgcttcagaccatcaaccgcttcattgtccgcccggtcacgccggacgaactgg<br>cagaacgcctgcatgaaaaacaggaactgccgccagtacggttaaaaacacaatcatattcgctgcgtctggaatga |
| *psu*<br><br>atggaaagcacagccttacagcaggcctttgacacctgtcagaataacaaagcagcatggctgcaacgcaaaaatgagct<br>ggcagcggccgaacaggaatatctgcggcttctgtcaggagaaggcagaaacgtcagtcgcctggacgaattacgcaata<br>ttatcgaagtcagaaaatggcaggtgaatcaggccgccggtcgttatattcgttcgcatgaagccgttcagcacatcagcatcc<br>gcgaccggctgaatgattttatgcagcagcacggcacagcactggcggccgcactggcaccggagctgatgggctacagt<br>gagctgacggccattgcccgaaactgtgccatacagcgtgccacagatgccctgcgtgaagcccttctgtcctggcttgcgaa<br>gggtgaaaaaattaattattccgcacaggatagcgacattttaacgaccatcggattcaggcctgacgtggcttcggtggatga<br>cagccgtgaaaaattcacccctgcgcagaacatgatttttcgcgtaaaagtgcgcaactggcatcacgtcagtcagtgtaa |
| Tsid terminator<br><br>aaagccatgcaccttatcgatgcatggctttttcagtaaaaacgggcggattttcggggaatt |
| P4 *cos* site (packaging signal)<br><br>agacactgacaagaaccaccttacatcaatctatttttgcatatttttcaggatagtgccatcaaaaaagacagttttggaccat<br>aggccatagattttcttcattatgctccaatgtattaacgaggtatgtactgaaggcactttgactacacaaataattaactaacttg<br>tcggtttgcgcgcggtgctttccccgcctcgcccgcccgctttgcggggcggttttaatgcagttgcactgacacgctcagaccg<br>cgccgggaatggcgcggtttgcagaaaatgaggcaggaaaacgcat |
| *crr*<br><br>ccgaaggtgaacaatccactgttcacccttcaccgtatattcacccgttatcacactgaaattaaaagagaaaaacgaaaggt<br>gaacagtgtgaacaatcaaatcaaaaaaaactttttcttcctgagtgatttcagtgcggaggattaatcaccggtatgagtcac<br>atcggcagaatgccggaggtgaagaatcgaatgttcacccttcacccattattcaccacctatcactctgaaataaaaggaga<br>aaacagaaaggtgaacagtgtgaacagttctttcgaaaaaaaatttttt |
| Promoter sequence for tracrRNA<br><br>agtattaagtattgttttatggctgataaatttctttgaatttctccttgattatttgttataaaagttataaaataatcttgtt |
| tracrRNA<br><br>ggaaccattcaaaacagcatagcaagttaaaataaggctagtccgttatcaacttgaaaaagtggcaccgagtcggtgc |
| Promoter and RBS sequence for *cas9* |

atagaatgataacaaaataaactactttttaaaagaattttgtgttataatctatttattattaagtattgggtaatattttttgaagaga
tattttgaaaaagaaaaattaaagcatattaaactaatttcggaggtcattaaaactattattgaaatcatcaaactcattatggat
ttaatttaaactttttattttaggaggcaaaa

*cas9*

atggataagaaatactcaataggcttagatatcggcacaaatagcgtcggatgggcggtgatcactgatgaatataaggttcc
gtctaaaaagttcaaggttctgggaaatacagaccgccacagtatcaaaaaaaatcttataggggctcttttatttgacagtgg
agagacagcggaagcgactcgtctcaaacggacagctcgtagaaggtatacacgtcggaagaatcgtatttgttatctacag
gagatttttcaaatgagatggcgaaagtagatgatagtttctttcatcgacttgaagagtctttttggtggaagaagacaagaa
gcatgaacgtcatcctatttttggaaatatagtagatgaagttgcttatcatgagaaatatccaactatctatcatctgcgaaaaa
aattggtagattctactgataaagcggatttgcgcttaatctatttggccttagcgcatatgattaagtttcgtggtcatttttgattga
gggagatttaaatcctgataatagtgatgtggacaaactatttatccagttggtacaaacctacaatcaattatttgaagaaaac
cctattaacgcaagtggagtagatgctaaagcgattctttctgcacgattgagtaaatcaagacgattagaaaatctcattgctc
agctccccggtgagaagaaaaatggcttatttgggaatctcattgctttgtcattgggtttgacccctaattttaaatcaaattttgat
ttggcagaagatgctaaattacagctttcaaaagatacttacgatgatgatttagataatttattggcgcaaattggagatcaata
tgctgatttgtttttggcagctaagaatttatcagatgctattttactttcagatatcctaagagtaaatactgaaataactaaggctc
ccctatcagcttcaatgattaaacgctacgatgaacatcatcaagacttgactcttttaaaagctttagttcgacaacaacttcca
gaaaagtataaagaaatcttttttgatcaatcaaaaaacggatatgcaggttatattgatgggggagctagccaagaagaattt
tataaatttatcaaaccaattttagaaaaaatggatggtactgaggaattattggtgaaactaaatcgtgaagatttgctgcgca
agcaacggacctttgacaacggctctattccccatcaaattcacttgggtgagctgcatgctattttgagaagacaagaagact
tttatccatttttaaaagacaatcgtgagaagattgaaaaaatcttgacttttcgaattccttattatgttggtccattggcgcgtggc
aatagtcgttttgcatggatgactcggaagtctgaagaaacaattaccccatggaattttgaagaagttgtcgataaaggtgctt
cagctcaatcatttattgaacgcatgacaaactttgataaaaatcttccaaatgaaaaagtactaccaaaacatagtttgctttat
gagtattttacggtttataacgaattgacaaaggtcaaatatgttactgaaggaatgcgaaaaccagcatttctttcaggtgaac
agaagaaagccattgttgatttactcttcaaaacaaatcgaaaagtaaccgttaagcaattaaaagaagattatttcaaaaaa
atagaatgttttgatagtgttgaaatttcaggagttgaagatagatttaatgcttcattaggtacctaccatgatttgctaaaaattatt
aaagataaagatttttggataatgaagaaaatgaagatatcttagaggatattgttttaacattgaccttatttgaagatagggga
gatgattgaggaaagacttaaaacatatgctcacctctttgatgataaggtgatgaaacagcttaaacgtcgccgttatactggt
tggggacgtttgtctcgaaaattgattaatggtattagggataagcaatctggcaaaacaatattagatttttgaaatcagatggt
tttgccaatcgcaattttatgcagctgatccatgatgatagtttgacatttaaagaagacattcaaaaagcacaagtgtctggac
aaggcgatagtttacatgaacatattgcaaatttagctggtagccctgctattaaaaaaggtattttacagactgtaaaagttgttg
atgaattggtcaaagtaatggggcggcataagccagaaaatatcgttattgaaatggcacgtgaaaatcagacaactcaaa
agggccagaaaaattcgcgagagcgtatgaaacgaatcgaagaaggtatcaaagaattaggaagtcagattcttaaaga
gcatcctgttgaaaatactcaattgcaaaatgaaaagctctatctctattatctccaaaatggaagagacatgtatgtggaccaa
gaattagatattaatcgtttaagtgattatgatgtcgatcacattgttccacaaagtttccttaaagacgattcaatagacaataag
gtcttaacgcgttctgataaaaatcgtggtaaatcggataacgttccaagtgaagaagtagtcaaaaagatgaaaaactattg
gagacaacttctaaacgccaagttaatcactcaacgtaagtttgataatttaacgaaagctgaacgtggaggtttgagtgaact
tgataaagctggttttatcaaacgccaattggttgaaactcgccaaatcactaagcatgtggcacaaattttggatagtcgcatg
aatactaaatacgatgaaaatgataaacttattcgagaggttaaagtgattaccttaaaatctaaattagtttctgacttccgaaa
agatttccaattctataaagtacgtgagattaacaattaccatcatgcccatgatgcgtatctaaatgccgtcgttggaactgcttt
gattaagaaatatccaaaacttgaatcggagtttgtctatggtgattataaagtttatgatgttcgtaaaatgattgctaagtctgag
caagaaataggcaaagcaaccgcaaaatatttcttttactctaatatcatgaacttcttcaaaacagaaattacacttgcaaatg
gagagattcgcaaacgccctctaatcgaaactaatggggaaactggagaaattgtctgggataaagggcgagattttgcca
cagtgcgcaaagtattgtccatgccccaagtcaatattgtcaagaaaacagaagtacagacaggcggattctccaaggagt
caattttaccaaaaagaaattcggacaagcttattgctcgtaaaaaagactgggatccaaaaaaatatggtggttttgatagtc
caacggtagcttattcagtcctagtggttgctaaggtggaaaaagggaaatcgaagaagttaaaatccgttaaagagttacta
gggatcacaattatggaaagaagttcctttgaaaaaaatccgattgactttttagaagctaaaggatataaggaagttaaaaa
agacttaatcattaaactacctaaatatagtctttttgagttagaaaacggtcgtaaacggatgctggctagtgccggagaatta
caaaaaggaaatgagctggctctgccaagcaaatatgtgaatttttttatatttagctagtcattatgaaaagttgaagggtagtcc
agaagataacgaacaaaaacaattgtttgtggagcagcataagcattatttagatgagattattgagcaaatcagtgaattttct
aagcgtgttattttagcagatgccaatttagataaagttcttagtgcatataacaaacatagagacaaaccaatacgtgaacaa
gcagaaaatattattcatttatttacgttgacgaatcttggagctcccgctgcttttaaatattttgatacaacaattgatcgtaaacg
atatacgtctacaaaagaagttttagatgccactcttatccatcaatccatcactggtctttatgaaacacgcattgatttgagtca
gctaggaggtgactga

crRNA leader sequence (in red), direct repeats (in blue) and spacer sequence. BsaI sites are underlined.

tatttcttaataactaaaaatatggtataatactcttaataaatgcagtaatacaggggcttttcaagactgaagtctagctgagac
aaatagtgcgattacgaaatttttagacaaaaatagtctacgaggttttagagctatgctgttttgaatggtcccaaaactgaga
ccagtctcggaagctcaaaggtctcgttttagagctatgctgttttgaatggtcccaaaac

Vector backbone with pBBR1 origin of replication and chloramphenicol resistance gene (*cat* in blue).

acccagctttcttgtacaaagtggttgatccggatctttgatcgggcacgtaagaggttccaactttcaccataatgaaataagat
cactaccgggcgtatttttgagttatcgagattttcaggagctaaggaagctaaaatggagaaaaaaatcactggatataccaccgttgatatatcccaatggcatcgtaaagaacattttgaggcatttcagtcagttgctcaatgtacctataaccagaccgttcag
ctggatattacggcctttttaaagaccgtaaagaaaaataagcacaagttttatccggcctttattcacattcttgcccgcctgatg
aatgctcatccggaatttcgtatggcaatgaaagacggtgagctggtgatatgggatagtgttcacccttgttacaccgttttccat
gagcaaactgaaacgttttcatcgctctggagtgaataccacgacgatttccggcagtttctacacatatattcgcaagatgtgg
cgtgttacggtgaaaacctggcctatttccctaaagggtttattgagaatatgtttttcgtctcagccaatccctgggtgagtttcac
cagttttgatttaaacgtggccaatatggacaacttcttcgcccccgttttcaccatgggcaaatattatacgcaaggcgacaag
gtgctgatgccgctggcgattcaggttcatcatgccgtttgtgatggcttccatgtcggcagaatgcttaatgaattacaacagta
ctgcgatgagtggcagggcggggcgtaatttgatatcgagctcgcttgcatttgacttttgtccttttccgctgcataaccctgcttc
ggggtcattatagcgattttttcggtatatccatcctttttcgcacgatatacaggattttgccaaagggttcgtgtagactttccttggt
gtatccaacggcgtcagccgggcaggataggtgaagtaggcccacccgcgagcgggtgttccttcttcactgtcccttattcgc
acctggcggtgctcaacgggaatcctgctctgcgaggctggccgtaggccggccctaccggcgcggcagcgttacccgtgt
cggcggctccaacggctcgccatcgtccagaaaacacggctcatcgggcatcggcaggcgctgctgcccgcgccgttccc
attcctccgtttcggtcaaggctggcaggtctggttccatgcccggaatgccgggctggctgggcggctcctcgccggggccg
gtcggtagttgctgctcgcccggatacagggtcgggatgcggcgcaggtcgccatgccccaacagcgattcgtcctggtcgtc
gtgatcaaccaccacggcggcactgaacaccgacaggcgcaactggtcgcggggctggccccacgccacgcggtcattg
accacgtaggccgacacggtgccggggccgttgagcttcacgacggagatccagcgctcggccaccaagtccttgactgc
gtattggaccgtccgcaaagaacgtccgatgagcttggaaagtgtcttctggctgaccaccacggcgttctggtggcccatctg
cgccacgaggtgatgcagcagcattgccgccgtgggtttcctcgcaataagcccggcccacgcctcatgcgctttgcgttccg
tttgcacccagtgaccgggcttgttcttggcttgaatgccgatttctctggactgcgtggccatgcttatctccatgcggtaggggtg
ccgcacggttgcggcaccatgcgcaatcagctgcaacttttcggcagcgcgacaacaattatgcgttgcgtaaaagtggcag
tcaattacagattttctttaacctacgcaatgagctattgcggggggtgccgcaatgagctgttgcgtacccccctttttttaagttgtt
gatttttaagtctttcgcatttcgccctatatctagttctttggtgcccaaagaagggcacccctgcggggttcccccacgccttcgg
cgcggctcccccctccggcaaaaagtggcccctccggggcttgttgatcgactgcgcggccttcggccttgcccaaggtggcg
ctgccccccttggaacccccgcactcgccgccgtgaggctcggggggcaggcgggcgggcttcgcccttcgactgcccccac
tcgcataggcttgggtcgttccaggcgcgtcaaggccaagccgctgcgcggtcgctgcgcgagccttgacccgccttccactt
ggtgtccaaccggcaagcgaagcgcgcaggccgcaggccggaggcttttccccagagaaaattaaaaaaattgatgggg
caaggccgcaggccgcgcagttggagccggtgggtatgtggtcgaaggctgggtagccggtgggcaatccctgtggtcaa
gctcgtgggcaggcgcagcctgtccatcagcttgtccagcagggttgtccacgggccgagcgaagcgagccagccggtgg
ccgctcgcggccatcgtccacatatccacgggctggcaagggagcgcagcgaccgcgcagggcgaagcccggagagc
aagcccgtagggggggcgcgcccagctgtctag

DNA sequence for plasmid encoding tail fiber and its chaperone protein

P2 late promoter sequence ($P_V$)

atgttgtctggtagttctacaaatgaatccagatagcataacttttatatattgtgcaatctcacatgc

P2 WT tail fiber (*H*)

atgagcataaaattcagaaccgttatcaccactgccggtgcagcaaagctggcagcggcaaccgcgccgggaaggcgga
aggtcggcattaccacgatggccgtcggggatggcggtggtaaattgcctgtcccggatgccggacagaccgggcttatcca

tgaagtctggcgacatgcgctgaacaaaatcagccaggacaaacgaaacagtaattatattatcgccgagctggttattccg
ccggaggtgggcggtttctggatgcgtgagcttggcctgtacgatgatgcgggaacgttaattgccgtggcgaacatggccga
aagctataagccagcccttgccgaaggctcaggacgttggcagacctgtcgcatggtcatcatcgtcagcagtgtggcctca
gtggagctgaccattgacaccacaacggtgatggcgacgcaggattacgttgatgacaaaattgcagagcacgaacagtc
acgacgtcacccggacgcctcgctgacagcaaaaggttttactcagttaagcagtgcgaccaacagcacgtctgaaacact
ggccgcaacgccgaaagcggtaaaggccgcgtatgacctggctaacgggaaatataccgcacaggacgccaccacag
cgcgaaaaggccttgtccagcttagtagcgccaccaacagcacgtctgaaacgctcgccgcaacaccaaaagccgttaag
acggtaatggatgaaacgaacaaaaaagcgccattaaacagccctgcactgaccggaacgccaacgacgccaactgcg
cgacagggaacgaataatactcagatcgcaaacacggctttcgttatggccgcgattgccgcccttgtagactcgtcgcctga
cgcactgaatacgctgaacgagctggcggcggcgctgggcaatgacccgaattttgctaccaccatgactaatgcgcttgcg
ggtaagcaaccgaaagatgctaccctgacggcgctggcggggcttgctactgcggcagacaggtttccgtattttacgggga
atgatgttgccagcctggcgaccctgacaaaagtcgggcgggatattctggctaaatcgaccgttgccgccgttatcgaatatc
tcggtttacaggaaacggtaaaccgagccgggaacgccgtgcaaaaaaatggcgataccttgtccggtggacttacttttga
aaacgactcaatccttgcctggattcgaaatactgactgggcgaagattggatttaaaaatgatgccgatggtgacactgattc
atacatgtggtttgaaacgggggataacggcaatgaatatttcaaatggagaagccgccagagtaccacaacaaaagacc
tgatgacgttgaaatgggatgcactaaatattcttgttaatgccgtcattaatggctgttttggagttggtacgacgaatgcactag
gtggtagctctattgttcttggtgataatgataccggatttaaacagaatggagacggtattcttgatgtttatgctaacagtcagcg
tgtattccgttttcagaatggagtggctattgcttttaaaaatattcaggcaggtgatagtaaaaagttctcgctatccagctctaat
acatccacgaagaatattacctttaatttatggggtgcttccacccgtccagtggttgcagagttaggcgatgaggccggatgg
catttctatagccagcgaaatacagataactcggtaatatttgctgttaacggtcagatgcaacccagcaactggggaaattttg
attcccgctatgtgaaagatgttcgcctgggtacgcgagttgttcaattgatggcgcgaggtggtcgttatgaaaaagccggac
acacgattaccggattaagaatcattggtgaagtagatggcgatgatgaagccatcttcaggccgatacaaaaatacatcaa
tggcacatggtataacgttgcgcaggtgtaa

P2 tail fiber chaperone protein (*G*)

Atgcagcatttaaagaacattaagtcaggtaatccaaaaacaaaagagcaatatcagctaacaaagaattttgatgttatctg
gttatggtccgaagacggaaaaaactggtatgaggaagtgaagaactttcagccagacacaataaagattgtttacgatgaa
aataatattattgtcgctatcaccagagatgcttcaacgcttaatcctgaaggttttagcgttgttgaggttcctgatattacctccaa
ccgacgtgctgacgactcaggtaaatggatgtttaaggatggtgctgtggttaaacggatttatacggcagatgaacagcaac
aacaggcagaatcacaaaaggccgcgttactttccgaagcggaaaacgttattcagccactggaacgcgctgtcaggctga
atatggcgacggatgaggaacgtgcacgactggagtcatgggaacgttacagcgttctggtcagccgtgtggatcctgcaaa
tcctgaatggccggaaatgccgcaataa

*P2- P1(S')* chimeric tail fiber (*P1(S')* region highlighted in blue)

atgagcataaaattcagaaccgttatcaccactgccggtgcagcaaagctggcagcggcaaccgcgccgggagggcgga
aggtcaacattaccacgatggccgtcggggatggcggtggtaaattgcctgtcccggatgccggacagaccggcttatcca
tgaagtctggcgacatgcgctgaacaaaatcagccaggacaaacgaaacagtaattatattatcgccgagctggttattccg
ccggaggtgggcggtttctggatgcgtgagcttggcctgtacgatgatgcgggaacgttaattgccgtggcgaacatggccga
aagctataagccagcccttgccgaaggctcaggacgttcgcagacctgtcgcatggtcatcatcgtcagcagtgtggcctca
gtggagctgaccattgacaccacaacggtgatggcgacgcaggattacgttgatgacaaaattgcagagcacgaacagtc
acgacgtcacccggacgcctcgctgacagcaaaaggttttactcagttaagcagtgcgaccaacagcacgtctgaaacact
ggccgcaacgccgaaagcggtaaaggccgcgtatgacctggctaacgggaaatataccgcacaggacgccaccacag
cgcgaaaaggccttgtccagcttagtagcgccaccaacagcacgtctgaaacgctcgccgcaacaccaaaagccgttaag
acggtaatggatgaaacgaacaaaaaagcgccattaaacagccctgcactgaccggaacgccaacgacgccaactgcg
cgacagggaacgaataatactcagatcgcaaacacggctttcgttatggccgcgattgccgcccttgtagactcgtcgcctga
cgcactgaatacgctgaacgagctggcggcggcgctgggcaatgacccgaattttgctaccaccatgactaatgcgcttgcg
ggtaagcaaccgaaagatgctaccctgacggcgctggcggggcttgctactgcggcagacaggtttccgtattttacgggga
atgatgttgccagcctggcgaccctgacaaaagtcgggcgggatattctggctaaatcgagcattcttgctgttatccaatacctt
ggtttaagagaactcggtaccagcggtgaaaagatcccccctgttgagcacggctaacacatggagtgcacgccagactttca
acggcgggatcaccggggcgctgacagggaacgccgataccgcaacgaaattgaaaacagccagaaacattaatggc
gtcaggttcgatggttctggtgacattaatatcaatactctggtatcgcgcggtcgcgtaacggccctggaggcgaatgcacag
ggaacatccgggattcagctgtatgaggcatacaacaatggctacccttccccctatggcaatgtgcttcaccttaaaggtgcc

accgctgctggcgaaggtgagttattcattggctggagtggcacgagcggtgcccatgcgcccgtacatatccgttcgcggcg
ggatactgattctgccaactggtctgaatgggcgcaggtctatacgtcaaaagattcaattcccggcgtcaatgccaaagggg
atcaggatacctctggtaatgcggctacagcgaccaagttgcagacagcatgtactatcaacggtgtctcgtttgatggttctaa
aaatattgagctaacggctgaaaatttaaatcttgagcgaacagtagaattagccgctgggtcattgcagaaaaatcagaac
ggcgcggatattcctggaaaagataccttcacaaaaaatattggtgcatgtcgcgcttttcacagttctattagtacaggtgcag
ggaactggacaacggcacaattgattgaatggctggattctcaaggggcattcaatcacccatactggatgtgcaaatgttcat
ggtcgtacggcaataataaaattataaccgatactggctgtggaactattcatcttgcaggttgcgttattgaggttatgggtaata
aaggtgccatgaccatccgtgtaacaacaccaagcacttccagcggtggcggaatcactaacgctcaattcacttatattaat
catggtgatgcttacgctcctggctggcgacgagactacaacacgaaaaacctgcaacctgcatttgctttagggcagacag
gaaacagggttgcaaatgataaagctgttggctggaactggaatagcggtgtttatgatgcagacctaaaaggcgcatcaac
attaattcttcatttcaatatgaacgcgggtagctgcccggctgtacaattacgcgtgaattataagaacggcggtatttattatcgt
tcagcgcgtgatggttatggatttgaggctgactggtcagagttttacaccacaacccgcaaaccctctgcgggggatgttggt
gcatatacgcaggcagaatgtaactcaaggtttattacaggtattcgcctgggcggtctgtcatctgtccagacatggaatggc
cccggctggtctgacaggtcaggttatgtcgttacgggttcagttaacgggaaccgtgatgaattaattgatacaacacaggca
aggccaattcagtattgcattaatgggacgtggtataacgcggggagtatttaa

P1 *U'* tail fiber chaperone protein

atgatgcacttaagaaatattacagctggcaaccctaaaacaaaagagcaataccagctaacgaaacaatttaacatcaaa
tggctttatacagaggatggaaaaaactggtatgaggaacaaaagaatttccagtatgatacgttgaaaatggcctatgacca
caacggcgttattatttgtattgaaaaggatgtttcagcaattaatccagaaggcgcaagcgtcgttgaattacctgatattacag
caaatcgccgggctgatatttctggtaaatggatgttcaaagatggcgtagtggtaaagcgaacttataccgaggaagagca
gaggcaacaagcggaaaatgaaaagcaaagtctgctacagctcgtcagggataaaacccagctatgggactcacagcta
cggctgggtatcatttccgccgagaataagcagaaattaaccgagtggatgctctttgcgcagaaagtcgaatccacagaca
cctccagcctaccagtaacatttcccgaacaacctgaatga

*P2-ϕV10* tail fiber (*ϕV10* region highlighted in blue)

atgagcataaaattcagaaccgttatcaccactgccggtgcagcaaagctggcagcggcaaccgcgccgggaaggcgga
aggtcggcattaccacgatggccgtcggggatggcggtggtaaattgcctgtcccggatgccggacagaccgggcttatcca
tgaagtctggcgacatgcgctgaacaaaatcagccaggacaaacgaaacagtaattatattatcgccgagctggttattccg
ccggaggtgggcggtttctggatgcgtgagcttggcctgtacgatgatgcgggaacgttaattgccgtggcgaacatggccga
aagctataagccagcccttgccgaaggctcaggacgttggcagacctgtcgcatggtcatcatcgtcagcagtgtggcctca
gtggagctgaccattgacaccacaacggtgatggcgacgcaggattacgttgatgacaaaattttagcggcaaatgatggat
ttaagtttattggtcagtgccctgatatattgacactaagaacaatagaacctgaaaaaaatggtcaaaggatcacgttgcgac
agcatacgatcggaactgggcttggtggaggggtattcagagctgtacttgatggaactggctataccgatgatgatggtgttgt
aataaaaacagcaggtggttctgtatggttacgggttaatgcagataaggttaatccgtttatgtttggggccactggcgtggctg
atgatacagcagcactgcaaaagatgcttgaatgtggcagggcggctgagcttggcacaaatgtctggaaagcatctaatctt
gagctaaataacaagtcgtgttcactatctgggtctggtctacacgtttccaggatagagcaaatatctggggcaacaggcgc
gttactgacaattactcaggattgcagcctgatttatttgtccgattgtggcctgtatggggatggaataactgcgggaacaagc
ggggtaacgatggagacaggaaacccaggtggggcaccaagctatccattcaataccgcccctgacgttcgtagggatcttt
acattagcaatgttcacattacaggatttgacgagttaggatttgattatcctgaaacgaatttttccgtctcaacgcatggattattc
ataagaaacattaaaaaaaccggggctaagatcggaactaccgattttacatggacaaaccttcaaattgatacgtgtgggc
aggaatgcttagtgcttgatggtgctggtaattgcagaattataggtgccaaattaatttgggctggttcagaaaatgaaacacc
atattcaggattacgtatttctaattcacaaaatgtaaacatgacaggagttgagttacaggactgtgcatacgatggattgtatat
taaaaattcaactgttgctatcagtgggttgaatactaacagaaatagtgcatcatcaaatctgtcataccataatatggtttttga
aaacagtatagtaactgtagatggttatgtttgccgtaactatgctgctacatcgctatatgatctaaattcacaagccggaaatg
tgaggtgtattggttccgatagtaccgtattgataaatggtatttacgagtcagaagtaaacagcgaacgactcatgggtgaca
ataatcttattcagccatattctggtgatttaataataaatggcctaaaaaactattatacatatacaggaagcgtaaaaaacaat
attcctacatttgacggagtcgttacaaccgcaacatatgtaagcgctccttcgatactagggcaaggaaacatgcttaagtta
acgcaatcaaacaaggataagttattattttctgataaagtatcgagacatgggtgtacaatcggccttgttctaatcccgtcattt
acgggtgcaacaactatgacagctttcactctggggagtggatattcgccctctggaaactctgctgttatgcaatttatagttaa
ctccagtggtgtgcagactatagcaatattattatccggtgatggtataactcaaacgttaacaagcgatctgacaacggagca
agcgttagcttcaggaggcgtatatcattttgcgatgggattcgctccaggccgattatggtggtctattattgatataaataccgg

aaggcgtatacgacgggcgtacagacaaccagatcttcacgccgcatttaatagcatattcaacagcggaacatcttctatca
ccgcattttcaggaccattagcaggtgatattgcatgtgaaggggcaggttcgcatgtttatgttggcggtttctcatctgagtcag
actacgcagcgagcagaatgtacggattatttaccccagtagacctggataaacaatactcattccgcacgctgaacggaaa
tatttaa

T7 terminator

ctgctaacaaagcccgaaaggaagctgagttggctgctgccaccgctgagcaataactagcataaccccttggggcctcta
aacgggtcttgaggggttttttgctgaaaggaggaactatatccggat

Vector backbone 4A3 with pSC101 origin of replication and ampicillin resistance gene (*bla* highlighted in blue).

ctctagaagcggccgcgaattccagaaatcatccttagcgaaagctaaggattttttttatctgaaattctgcctcgtgatacgcct
atttttataggttaatgtcatgataataatggtttcttagacgtcaggtggcaccatgggaaatgtgcgcggaacccctatttgtttat
ttttctaaatacattcaaatatgtatccgctcatgagacaataaccctgataaatgcttcaataatattgaaaaaggaagagtatg
agtattcaacatttccgtgtcgcccttattcccttttttgcggcattttgccttcctgtttttgctcacccagaaacgctggtgaaagtaa
aagatgctgaagatcagttgggtgcacgagtgggttacatcgaactggatctcaacagcggtaagatccttgagagttttcgcc
ccgaagaacgttttccaatgatgagcacttttaaagttctgctatgtggcgcggtattatcccgtattgacgccgggcaagagca
actcggtcgccgcatacactattctcagaatgacttggttgagtactcaccagtcacagaaaagcatcttacggatggcatgac
agtaagagaattatgcagtgctgccataaccatgagtgataacactgcggccaacttacttctgacaacgatcggaggaccg
aaggagctaaccgcttttttgcacaacatgggggatcatgtaactcgccttgatcgttgggaaccggagctgaatgaagccat
accaaacgacgagcgtgacaccacgatgcctgtagcaatggcaacaacgttgcgcaaactattaactggcgaactacttac
tctagcttcccggcaacaattaatagactggatggaggcggataaagttgcaggaccacttctgcgctcggcccttccggctg
gctggtttattgctgataaatctggagccggtgagcgtgggtctcgcggtatcattgcagcactggggccagatggtaagccct
cccgtatcgtagttatctacacgacggggagtcaggcaactatggatgaacgaaatagacagatcgctgagataggtgcctc
actgattaagcattggtaactgtcagaccaagtttacgagctcgcttggactcctgttgatagatccagtaatgacctcagaact
ccatctggatttgttcagaacgctcggttgccgccgggcgttttttattggtgagaatccaagcactagggacagtaagacgggt
aagcctgttgatgataccgctgccttactgggtgcattagccagtctgaatgacctgtcacgggataatccgaagtggtcagac
tggaaaatcagagggcaggaactgctgaacagcaaaaagtcagatagcaccacatagcagacccgccataaaacgccc
tgagaagcccgtgacgggcttttcttgtattatgggtagtttccttgcatgaatccataaaaggcgcctgtagtgccatttaccccc
attcactgccagagccgtgagcgcagcgaactgaatgtcacgaaaaagacagcgactcaggtgcctgatggtcggagac
aaaaggaatattcagcgatttgcccgagcttgcgagggtgctacttaagcctttagggttttaaggtctgttttgtagaggagcaa
acagcgtttgcgacatccttttgtaatactgcggaactgactaaagtagtgagttatacacagggctgggatctattctttttatctttt
tttattctttctttattctataaattataaccacttgaatataaacaaaaaaaacacacaaaggtctagcggaatttacagagggtc
tagcagaatttacaagttttccagcaaaggtctagcagaatttacagatacccacaactcaaaggaaaaggacatgtaattat
cattgactagcccatctcaattggtatagtgattaaaatcacctagaccaattgagatgtatgtctgaattagttgttttcaaagca
aatgaactagcgattagtcgctatgacttaacggagcatgaaaccaagctaattttatgctgtgtggcactactcaaccccacg
attgaaaaccctacaaggaaagaacggacggtatcgttcacttataaccaatacgctcagatgatgaacatcagtagggaa
aatgcttatggtgtattagctaaagcaaccagagagctgatgacgagaactgtggaaatcaggaatcctttggttaaaggcttt
gagattttccagtggacaaactatgccaagttctcaagcgaaaaattagaattagtttttagtgaagagatattgccttatcttttcc
agttaaaaaaattcataaaatataatctggaacatgttaagtcttttgaaaacaaatactctatgaggatttatgagtggttattaa
aagaactaacacaaaagaaaactcacaaggcaaatatagagattagccttgatgaatttaagttcatgttaatgcttgaaaat
aactaccatgagtttaaaaggcttaaccaatgggttttgaaaccaataagtaaagatttaaacacttacagcaatatgaaattg
gtggttgataagcgaggccgcccgactgatacgttgattttccaagttgaactagatagacaaatggatctcgtaaccgaactt
gagaacaaccagataaaaatgaatggtgacaaaataccaacaaccattacatcagattcctacctacgtaacggactaag
aaaaacactacacgatgctttaactgcaaaaattcagctcaccagttttgaggcaaaatttttgagtgacatgcaaagtaagca
tgatctcaatggttcgttctcatggctcacgcaaaaacaacgaaccacactagagaacatactggctaaatacggaaggatc
tgaggttcttatggctcttgtatctatcagtgaagcatcaagactaacaaacaaaagtagaacaactgttcaccgttagatatca
aagggaaaactgtccatatgcacagatgaaaacggtgtaaaaaagatagatacatcagagcttttacgagtttttggtgcattt
aaagctgttcaccatgaacagatcgacaatgtaacctcgagattaccgcctttgagtgagctgataccgctcgccgcagccg
aacgaccgagcgcagcgagtcagtgagcgaggaagcctgcataacgcgaagtaatcttttcggttttaaagaaaaagggc
agggtggtgacaccttgcccttttttgccggactgcagcggccgctactagtattatta

DNA sequences for full plasmids

P4 cosmid (Benchling link: https://benchling.com/s/seq-6yFP2RWWbSpLiQ5TUrlh?m=slm-wcMQes28bw1I34vy2zFb)

ctcggtaccaaattccagaaaagaggcctcccgaaagggggcctttttcgttttggtccgtcaagttgtcataattggtaacg
aatcagacaattgacggctcgatggagtagcatagagaagtcagagtagaatagaagtatcaaaaaaagcaccgactcg
gtgccactttttcaagttgataacggactagccttattttaacttgctatgctgttttgaatggttccaacaagattattttataacttttat
aacaaataatcaaggagaaattcaaagaaatttatcagccataaaacaatacttaatactatagaatgataacaaaataaac
tactttttaaaagaattttgtgttataatctatttattattaagtattgggtaatatttttgaagagatattttgaaaaagaaaaattaaa
gcatattaaactaatttcggaggtcattaaaactattattgaaatcatcaaactcattatggatttaatttaaactttttattttaggag
gcaaaaatggataagaaatactcaataggcttagatatcggcacaaatagcgtcggatgggcggtgatcactgatgaatata
aggttccgtctaaaaagttcaaggttctgggaaatacagaccgccacagtatcaaaaaaaatcttataggggctcttttatttga
cagtggagagacagcggaagcgactcgtctcaaacggacagctcgtagaaggtatacacgtcggaagaatcgtatttgttat
ctacaggagatttttcaaatgagatggcgaaagtagatgatagtttctttcatcgacttgaagagtctttttggtggaagaagac
aagaagcatgaacgtcatcctatttttggaaatatagtagatgaagttgcttatcatgagaaatatccaactatctatcatctgcg
aaaaaaattggtagattctactgataaagcggatttgcgcttaatctatttggccttagcgcatatgattaagtttcgtggtcatttttt
gattgagggagatttaaatcctgataatagtgatgtggacaaactatttatccagttggtacaaacctacaatcaattatttgaag
aaaaccctattaacgcaagtggagtagatgctaaagcgattctttctgcacgattgagtaaatcaagacgattagaaaatctc
attgctcagctccccggtgagaagaaaaatggcttatttgggaatctcattgctttgtcattgggtttgacccctaattttaaatcaa
attttgatttggcagaagatgctaaattacagctttcaaaagatacttacgatgatgatttagataatttattggcgcaaattggag
atcaatatgctgatttgtttttggcagctaagaatttatcagatgctattttactttcagatatcctaagagtaaatactgaaataacta
aggctcccctatcagcttcaatgattaaacgctacgatgaacatcatcaagacttgactcttttaaaagctttagttcgacaacaa
cttccagaaaagtataaagaaatcttttttgatcaatcaaaaaacggatatgcaggttatattgatgggggagctagccaagaa
gaattttataaatttatcaaaccaattttagaaaaaatggatggtactgaggaattattggtgaaactaaatcgtgaagatttgctg
cgcaagcaacggacctttgacaacggctctattccccatcaaattcacttgggtgagctgcatgctattttgagaagacaagaa
gacttttatccatttttaaaagacaatcgtgagaagattgaaaaaatcttgacttttcgaattccttattatgttggtccattggcgcgt
ggcaatagtcgttttgcatggatgactcggaagtctgaagaaacaattaccccatggaattttgaagaagttgtcgataaaggt
gcttcagctcaatcatttattgaacgcatgacaaactttgataaaaatcttccaaatgaaaaagtactaccaaaacatagtttgct
ttatgagtattttacggtttataacgaattgacaaaggtcaaatatgttactgaaggaatgcgaaaaccagcatttctttcaggtga
acagaagaaagccattgttgatttactcttcaaaacaaatcgaaaagtaaccgttaagcaattaaaagaagattatttcaaaa
aaatagaatgttttgatagtgttgaaatttcaggagttgaagatagatttaatgcttcattaggtacctaccatgatttgctaaaaatt
attaaagataaagatttttttggataatgaagaaaatgaagatatcttagaggatattgttttaacattgaccttatttgaagatagg
gagatgattgaggaaagacttaaaacatatgctcacctctttgatgataaggtgatgaaacagcttaaacgtcgccgttatact
ggttggggacgtttgtctcgaaaattgattaatggtattagggataagcaatctggcaaaacaatattagatttttttgaaatcagat
ggttttgccaatcgcaattttatgcagctgatccatgatgatagtttgacatttaaagaagacattcaaaaagcacaagtgtctgg
acaaggcgatagtttacatgaacatattgcaaatttagctggtagccctgctattaaaaaaggtattttacagactgtaaaagttg
ttgatgaattggtcaaagtaatggggcggcataagccagaaaatatcgttattgaaatggcacgtgaaaatcagacaactca
aaagggccagaaaaattcgcgagagcgtatgaaacgaatcgaagaaggtatcaaagaattaggaagtcagattcttaaa
gagcatcctgttgaaaatactcaattgcaaaatgaaaagctctatctctattatctccaaaatggaagagacatgtatgtggacc
aagaattagatattaatcgtttaagtgattatgatgtcgatcacattgttccacaaagtttccttaaagacgattcaatagacaata
aggtcttaacgcgttctgataaaaatcgtggtaaatcggataacgttccaagtgaagaagtagtcaaaaagatgaaaaactat
tggagacaacttctaaacgccaagttaatcactcaacgtaagtttgataatttaacgaaagctgaacgtggaggtttgagtgaa
cttgataaagctggttttatcaaacgccaattggttgaaactcgccaaatcactaagcatgtggcacaaattttggatagtcgcat
gaatactaaatacgatgaaaatgataaacttattcgagaggttaaagtgattaccttaaaatctaaattagtttctgacttccgaa
aagatttccaattctataaagtacgtgagattaacaattaccatcatgcccatgatgcgtatctaaatgccgtcgttggaactgctt
tgattaagaaatatccaaaacttgaatcggagtttgtctatggtgattataaagtttatgatgttcgtaaaatgattgctaagtctga
gcaagaaataggcaaagcaaccgcaaaatatttcttttactctaatatcatgaacttcttcaaaacagaaattacacttgcaaat
ggagagattcgcaaacgccctctaatcgaaactaatggggaaactggagaaattgtctgggataaagggcgagattttgcc
acagtgcgcaaagtattgtccatgccccaagtcaatattgtcaagaaaacagaagtacagacaggcggattctccaaggag
tcaattttaccaaaaagaaattcggacaagcttattgctcgtaaaaaagactgggatccaaaaaaatatggtggttttgatagtc
caacggtagcttattcagtcctagtggttgctaaggtggaaaaagggaaatcgaagaagttaaaatccgttaaagagttacta
gggatcacaattatggaaagaagttcctttgaaaaaaatccgattgactttttagaagctaaaggatataaggaagttaaaaa
agacttaatcattaaactacctaaatatagtctttttgagttagaaaacggtcgtaaacggatgctggctagtgccggagaatta
caaaaaggaaatgagctggctctgccaagcaaatatgtgaatttttttatatttagctagtcattatgaaaagttgaagggtagtcc
agaagataacgaacaaaaacaattgtttgtggagcagcataagcattatttagatgagattattgagcaaatcagtgaattttct

aagcgtgttattttagcagatgccaatttagataaagttcttagtgcatataacaaacatagagacaaaccaatacgtgaacaa
gcagaaaatattattcatttatttacgttgacgaatcttggagctcccgctgcttttaaatattttgatacaacaattgatcgtaaacg
atatacgtctacaaaagaagttttagatgccactcttatccatcaatccatcactggtctttatgaaacacgcattgatttgagtca
gctaggaggtgactgaagtatattttagatgaagattatttcttaataactaaaaatatggtataatactcttaataaatgcagtaat
acaggggcttttcaagactgaagtctagctgagacaaatagtgcgattacgaaatttttttagacaaaaatagtctacgaggtttt
agagctatgctgttttgaatggtcccaaaactgagaccagtctcggaagctcaaaggtctcgttttagagctatgctgttttgaatg
gtcccaaaacttcagcacactgagacttgttgagtggatccgaggcttggattctcaccaataaaaaacgcccggcggcaac
cgagcgttctgaacaaatccagatggagttctgaggtcattactggatctatcaacaggagtcctagacagctgggcgcgccc
cccctacgggcttgctctccgggcttcgccctgcgcggtcgctgcgctcccttgccagcccgtggatatgtggacgatggccgc
gagcggccaccggctggctcgcttcgctcggcccgtggacaaccctgctggacaagctgatggacaggctgcgcctgccc
acgagcttgaccacagggattgcccaccggctacccagccttcgaccacatacccaccggctccaactgcgcggcctgcg
gccttgccccatcaattttttttaatttctctggggaaaagcctccggcctgcggcctgcgcgcttcgcttgccggttggacaccaa
gtggaaggcgggtcaaggctcgcgcagcgaccgcgcagcggcttggccttgacgcgcctggaacgacccaagcctatgc
gagtgggggcagtcgaagggcgaagcccgcccgcctgcccccgagcctcacggcggcgagtgcgggggttccaaggg
ggcagcgccaccttgggcaaggccgaaggccgcgcagtcgatcaacaagccccggaggggccactttttgccggaggg
ggagccgcgccgaaggcgtgggggaaccccgcaggggtgcccttctttgggcaccaaagaactagatatagggcgaaat
gcgaaagacttaaaaatcaacaacttaaaaaagggggtacgcaacagctcattgcggcacccccgcaatagctcattg
cgtaggttaaagaaaatctgtaattgactgccacttttacgcaacgcataattgttgtcgcgctgccgaaaagttgcagctgattg
cgcatggtgccgcaaccgtgcggcacccctaccgcatggagataagcatggccacgcagtccagagaaatcggcattcaa
gccaagaacaagcccggtcactgggtgcaaacggaacgcaaagcgcatgaggcgtgggccgggcttattgcgaggaaa
cccacggcggcaatgctgctgcatcacctcgtggcgcagatgggccaccagaacgccgtggtggtcagccagaagacact
ttccaagctcatcggacgttctttgcggacggtccaatacgcagtcaaggacttggtggccgagcgctggatctccgtcgtgaa
gctcaacggccccggcaccgtgtcggcctacgtggtcaatgaccgcgtggcgtggggccagccccgcgaccagttgcgcct
gtcggtgttcagtgccgccgtggtggttgatcacgacgaccaggacgaatcgctgttggggcatggcgacctgcgccgcatc
ccgaccctgtatccgggcgagcagcaactaccgaccggccccggcgaggagccgcccagccagcccggcattccgggc
atggaaccagacctgccagccttgaccgaaacggaggaatgggaacggcgcgggcagcagcgcctgccgatgcccgat
gagccgtgttttctggacgatggcgagccgttggagccgccgacacgggtaacgctgccgcgccggtagggccggcctac
ggccagcctcgcagagcaggattcccgttgagcaccgccaggtgcgaataagggacagtgaagaaggaacacccgctc
gcgggtgggcctacttcacctatcctgcccggctgacgccgttggatacaccaaggaaagtctacacgaaccctttggcaaa
atcctgtatatcgtgcgaaaaaggatggatataccgaaaaaatcgctataatgaccccgaagcagggttatgcagcggaaa
aggacaaaagtcaaatgcaagcgagctcgatatcaaattacgccccgccctgccactcatcgcagtactgttgtaattcatta
agcattctgccgacatggaagccatcacaaacggcatgatgaacctgaatcgccagcggcatcagcaccttgtcgccttgcg
tataatatttgcccatggtgaaaacgggggcgaagaagttgtccatattggccacgtttaaatcaaaactggtgaaactcaccc
agggattggctgagacgaaaaacatattctcaataaaccctttagggaaataggccaggttttcaccgtaacacgccacatct
tgcgaatatatgtgtagaaactgccggaaatcgtcgtggtattcactccagagcgatgaaaacgtttcagtttgctcatggaaa
acggtgtaacaagggtgaacactatcccatatcaccagctcaccgtctttcattgccatacgaaattccggatgagcattcatc
aggcgggcaagaatgtgaataaaggccggataaaacttgtgcttatttttctttacggtctttaaaaaggccgtaatatccagct
gaacggtctggttataggtacattgagcaactgactgaaatgcctcaaaatgttctttacgatgccattgggatatatcaacggt
ggtatatccagtgattttttttctccattttagcttccttagctcctgaaaatctcgataactcaaaaaatacgcccggtagtgatcttat
ttcattatggtgaaagttggaacctcttacgtgcccgatcaaagatccggatcaaccactttgtacaagaaagctgggtccgaa
ggtgaacaatccactgttcacccttcaccgtatattcacccgttatcacactgaaattaaaagagaaaaacgaaaggtgaac
agtgtgaacaatcaaatcaaaaaaaactttttttcttcctgagtgatttcagtgcggaggattaatcaccggtatgagtcacatcg
gcagaatgccggaggtgaagaatcgaatgttcacccttcacccattattcaccacctatcactctgaaataaaaggagaaaa
cagaaaggtgaacagtgtgaacagttctttcgaaaaaaaatttttttggcgcgtcagcgggtgttggcgggtgtcggggctggct
taactatgcggcatcagagcagattgtactgagagtgcaccatatgcggtgtgaaataccgcacagatgcgtaaggagaaa
ataccgcatcaggcgccattcgccattcaggctgcgcaactgttgggaagggcgatcggtgcgggcctcttcgctattacgcc
agctggcgaaagggggatgtgctgcaaggcgattaagttgggtaacgccagggttttcccagtcacgacgttgtaaaacgac
ggccagtgaaattattaacctaggctgacttcatccgcacacacgagccgcgccccttgcgcgacgggctggcggtattggc
ggcgctgaaagaaaccgcaagaattcttaaggatcttgcgacactgacaagaaccaccttacatcaatctattttttgcatatttt
tcaggatagtgccatcaaaaaagacagttttggaccataggccatagattttcttcattatgctccaatgtattaacgaggtatgt
actgaaggcactttgactacacaaataattaactaacttgtcggtttgcgcgcggtgctttccccgcctcgcccgcccgctttgcg
gggcggttttaatgcagttgcactgacacgctcagaccgcgccgggaatggcgcggtttgcagaaaatgaggcaggaaaa
cgcatgcaaagccatgcaccttatcgatgcatggctttttttcagtaaaaacgggcggattttcggggaattttacactgactgac
gtgatgccagttgcgcacttttacgcgaaaaaatcatgttctgcgcaggggtgaatttttcacggctgtcatccaccgaagccac
gtcaggcctgaatccgatggtcgttaaaatgtcgctatcctgtgcggaataattaatttttttcacccttcgcaagccaggacaga

agggcttcacgcagggcatctgtggcacgctgtatggcacagtttcgggcaatggccgtcagctcactgtagcccatcagctc
cggtgccagtgcggccgccagtgctgtgccgtgctgctgcataaaatcattcagccggtcgcggatgctgatgtgctgaacgg
cttcatgcgaacgaatataacgaccggcggcctgattcacctgccattttctgacttcgataatattgcgtaattcgtccaggcga
ctgacgtttctgccttctcctgacagaagccgcagatattcctgttcggccgctgccagctcatttttgcgttgcagccatgctgcttt
gttattctgacaggtgtcaaaggcctgctgtaaggctgtgctttccatcgttatctctttctcatcatgctgaagaataaaaatacgg
tgtgcggcgacggccggtgttaaccggcagccctcattccagacgcagcgaatatgattgtgtttttaaccgtactggcggcag
ttcctgtttttcatgcaggcgttctgccagttcgtccggcgtgaccgggcggacaatgaagcggttgatggtctgaagcgttttaa
acaccagaccacagcccggatccgtgcacacataaaaacgctcggtgacttcctgagacagacgccgcgatgttcttgaca
gtgcaaggcctttacatctccgacaacaatatccggtaacaagcattcttttcgggcgtttcataccgcaggaggctgacgtca
gtgaatcgcggtatctctgtttgcctgaaatgtattccattcctgaatctttactgtcagagaaaaagctttcactcgcttcaaatgtc
gcagagcaataaatattccggcactgtgcaatcattatcttggtgccatcgtccatgaaatgtgcgcgacgggtgtgagcaac
atgtccacacgacggacagtaaatcatgacagcagccctctggccttaagctctgctccctgctggtctattttgtcctgccaca
ccttgcgctgtgccggtgtgcctgccacctcataatccatgtgcgggagtgttgccgctgacagtccggtcagccggagaacc
ggctcgccggtgaggctgatttgcatctgtttaattttctgttccagcgatgatttcacctgctgcatgacagccttttccggtgcgac
gtagccctgatggccggtggtgttggcgagcggattttcctgtaccagaatgctcagatgcattgcccggacaagcgcctcac
aggtttcattcagggcgtgttccagctcatgctctgcatacagactcagaaggtgatgatgtgccttccggtaggcggtggccgt
gctgtcacacgcccctttcaggcgttcacgttcgaaattcagcaccacggccagattgtcatattcctgtaccagctcccggcgt
gccacgcgctcaatgtggcgctgtttcagctcgtcgctcaggacaccaccggctgcacgaaaggccgtgcgccagtcgtca
gcgtcgtttccgtcggcctgcgccagcgcatttttttcctgctctgcccgttcaatggccgtgacagtctcatccatcaggcgggc
gttctcaagatgggcggctctggccttttccagttgtgccagtgcgggttgcagatattcagggatagtgtggtcagacattttccg
gctcctcgtcacttcaggttaagaaaattgtgacgtacaccggacaacaacacgacgcattgcagatgtgccagccctgaca
caggagactcatcctcagaccggcaagccaggaaaaggtcgcaggaaaaaccggcttactgtttgtttttttatattttactgttc
acctctgttcaccttaataaaaaagataagtaatacagtaagttaaagggtgaacaatcgcagtaatgactgttcaccgtctgtt
caccactgttcacccgttgatgggctttttgtgctgtttactactgtttgtttttattaattcgccgggaatgaataagaagaaacgattt
gtatttcactataaaaattacaaattgctttaagtggctttaaatgactttaagtggagatgaacaaaaaacacacagccattgta
agacagcctgaacaaatccccccctgttgcgtctgctgaaaatattcacaaaataaagcgctcagcacaggcagcacatcat
catcaccaccactaactagcataaccccttggggcctctaaacgggtcttgaggggttttttgatcccaatggcgcgccgagctt
ggctcgagcatggtcatagctgtttcctgtgtgaaattgttatccgctcacaattccacacaacatacgagccggaagcataaa
gtgtaaagcctggggtgcctaatgagtgagctaactcacattaattgcgttgcgctcactgcccgctttccagtcgggaaacctg
tcgtgccagctgcattaatgaatcggccaacgcgcggggagaggcggtttgcgtattgggcgctcttccgcttcctcgctcact
gactcgctgcgctcggtcgttcggctgcggcgagcggtatcagctcactcaaaggcggtaatacggttatccacagaatcag

Plasmid expressing P2 tail fiber H and chaperone protein G (pP2_tail_fiber)
(Benchling link: https://benchling.com/s/seq-vNfYIKnwRP4OYpmSkkRo?m=slm-TrNcJk4I2QpwwQ3lBP3R)

tagctttcgctaaggatgatttctggaattcgcggccgcttctagagtacgctctccttgaatgttgtctggtagttctacaaatgaat
ccagatagcataacttttatatattgtgcaatctcacatgcatgagcataaaattcagaaccgttatcaccactgccggtgcagc
aaagctggcagcggcaaccgcgccgggagggcggaaggtcaacattaccacgatggccgtcggggatggcggtggtaa
attgcctgtcccggatgccggacagaccgggcttatccatgaagtctggcgacatgcgctgaacaaaatcagccaggacaa
acgaaacagtaattatattatcgccgagctggttattccgccggaggtgggcggtttctggatgcgtgagcttggcctgtacgat
gatgcgggaacgttaattgccgtggcgaacatggccgaaagctataagccagcccttgccgaaggctcaggacgttcgcag
acctgtcgcatggtcatcatcgtcagcagtgtggcctcagtggagctgaccattgacaccacaacggtgatggcgacgcagg
attacgttgatgacaaaattgcagagcacgaacagtcacgacgtcacccggacgcctcgctgacagcaaaaggttttactca
gttaagcagtgcgaccaacagcacgtctgaaacactggccgcaacgccgaaagcggtaaaggccgcgtatgacctggct
aacgggaaatataccgcacaggacgccaccacagcgcgaaaaggccttgtccagcttagtagcgccaccaacagcacgt
ctgaaacgctcgccgcaacaccaaaagccgttaagacggtaatggatgaaacgaacaaaaaagcgccattaaacagcc
ctgcactgaccggaacgccaacgacgccaactgcgcgacagggaacgaataatactcagatcgcaaacacggctttcgtt
atggccgcgattgccgcccttgtagactcgtcgcctgacgcactgaatacgctgaacgagctggcggcggcgctgggcaat
gacccgaattttgctaccaccatgactaatgcgcttgcgggtaagcaaccgaaagatgctaccctgacggcgctggcgggg
cttgctactgcggcagacaggtttccgtattttacggggaatgatgttgccagcctggcgaccctgacaaaagtcgggcggga
tattctggctaaatcgaccgttgccgccgttatcgaatatctcggtttacaggaaacggtaaaccgagccgggaacgccgtgc
aaaaaaatggcgataccttgtccggtggacttacttttgaaaacgactcaatccttgcctggattcgaaatactgactgggcga
agattggatttaaaaatgatgccgatggtgacactgattcatacatgtggtttgaaacgggggataacggcaatgaatatttca
aatggagaagccgccagagtaccacaacaaaagacctgatgacgttgaaatgggatgcactaaatattcttgttaatgccgt
cattaatggctgttttggagttggtacgacgaatgcactaggtggtagctctattgttcttggtgataatgataccggatttaaacag

| |
|---|
| aatggagacggtattcttgatgtttatgctaacagtcagcgtgtattccgttttcagaatggagtggctattgcttttaaaaatattca<br>ggcaggtgatagtaaaaagttctcgctatccagctctaatacatccacgaagaatattacctttaatttatggggtgcttccaccc<br>gtccagtggttgcagagttaggcgatgaggccggatggcatttctatagccagcgaaatacagataactcggtaatatttgctg<br>ttaacggtcagatgcaacccagcaactggggaaattttgattcccgctatgtgaaagatgttcgcctgggtacgcgagttgttca<br>attgatggcgcgaggtggtcgttatgaaaaagccggacacacgattaccggattaagaatcattggtgaagtagatggcgat<br>gatgaagccatcttcaggccgatacaaaaatacatcaatggcacatggtataacgttgcgcaggtgtaagttatgcagcattta<br>aagaacattaagtcaggtaatccaaaaacaaaagagcaatatcagctaacaaagaattttgatgttatctggttatggtccga<br>agacggaaaaaactggtatgaggaagtgaagaactttcagccagacacaataaagattgtttacgatgaaaataatattatt<br>gtcgctatcaccagagatgcttcaacgcttaatcctgaaggttttagcgttgttgaggttcctgatattacctccaaccgacgtgct<br>gacgactcaggtaaatggatgtttaaggatggtgctgtggttaaacggatttatacggcagatgaacagcaacaacaggcag<br>aatcacaaaaggccgcgttactttccgaagcggaaaacgttattcagccactggaacgcgctgtcaggctgaatatggcgac<br>ggatgaggaacgtgcacgactggagtcatgggaacgttacagcgttctggtcagccgtgtggatcctgcaaatcctgaatgg<br>ccggaaatgccgcaataaggatccgaattcgagctccgtcgacaagcttgcggccgcactcgagcaccaccaccaccacc<br>actgagatccggctgctaacaaagcccgaaaggaagctgagttggctgctgccaccgctgagcaataactagcataaccc<br>cttggggcctctaaacgggtcttgaggggttttttgctgaaaggaggaactatatccggattaataatactagtagcggccgctg<br>cagtccggcaaaaaagggcaaggtgtcaccaccctgcccttttttctttaaaaccgaaaagattacttcgcgttatgcaggcttc<br>ctcgctcactgactcgctgcgctcggtcgttcggctgcggcgagcggtatcagctcactcaaaggcggtaatctcgaggttaca<br>ttgtcgatctgttcatggtgaacagctttaaatgcaccaaaaactcgtaaaagctctgatgtatctatcttttttacaccgttttcatct<br>gtgcatatggacagttttccctttgatatctaacggtgaacagttgttctacttttgtttgttagtcttgatgcttcactgatagatacaa<br>gagccataagaacctcagatccttccgtatttagccagtatgttctctagtgtggttcgttgtttttgcgtgagccatgagaacgaa<br>ccattgagatcatgcttactttgcatgtcactcaaaaattttgcctcaaaactggtgagctgaatttttgcagttaaagcatcgtgta<br>gtgtttttcttagtccgttacgtaggtaggaatctgatgtaatggttgttggtattttgtcaccattcatttttatctggttgttctcaagttcg<br>gttacgagatccatttgtctatctagttcaacttggaaaatcaacgtatcagtcgggcggcctcgcttatcaaccaccaatttcata<br>ttgctgtaagtgtttaaatctttacttattggtttcaaaacccattggttaagccttttaaactcatggtagttattttcaagcattaacat<br>gaacttaaattcatcaaggctaatctctatatttgccttgtgagttttcttttgtgttagttcttttaataaccactcataaatcctcataga<br>gtatttgttttcaaaagacttaacatgttccagattatattttatgaatttttttaactggaaaagataaggcaatatctcttcactaaaa<br>actaattctaatttttcgcttgagaacttggcatagtttgtccactggaaaatctcaaagcctttaaccaaaggattcctgatttcca<br>cagttctcgtcatcagctctctggttgctttagctaatacaccataagcattttccctactgatgttcatcatctgagcgtattggttata<br>agtgaacgataccgtccgttctttccttgtagggttttcaatcgtggggttgagtagtgccacacagcataaaattagcttggtttca<br>tgctccgttaagtcatagcgactaatcgctagttcatttgctttgaaaacaactaattcagacatacatctcaattggtctaggtgat<br>tttaatcactataccaattgagatgggctagtcaatgataattacatgtccttttcctttgagttgtgggtatctgtaaattctgctaga<br>cctttgctggaaaacttgtaaattctgctagaccctctgtaaattccgctagacctttgtgtgttttttttgtttatattcaagtggttataat<br>ttatagaataaagaaagaataaaaaaagataaaaagaatagatcccagccctgtgtataactcactactttagtcagttccgc<br>agtattacaaaaggatgtcgcaaacgctgtttgctcctctacaaaacagaccttaaaaccctaaaggcttaagtagcaccctc<br>gcaagctcgggcaaatcgctgaatattccttttgtctccgaccatcaggcacctgagtcgctgtctttttcgtgacattcagttcgct<br>gcgctcacggctctggcagtgaatgggggtaaatggcactacaggcgccttttatggattcatgcaaggaaactacccataat<br>acaagaaaagcccgtcacgggcttctcagggcgttttatggcgggtctgctatgtggtgctatctgactttttgctgttcagcagtt<br>cctgccctctgattttccagtctgaccacttcggattatcccgtgacaggtcattcagactggctaatgcacccagtaaggcagc<br>ggtatcatcaacaggcttacccgtcttactgtccctagtgcttggattctcaccaataaaaaacgcccggcggcaaccgagcgt<br>tctgaacaaatccagatggagttctgaggtcattactggatctatcaacaggagtccaagcgagctcgtaaacttggtctgaca<br>gttaccaatgcttaatcagtgaggcacctatctcagcgatctgtctatttcgttcatccatagttgcctgactccccgtcgtgtagat<br>aactacgatacgggagggcttaccatctggccccagtgctgcaatgataccgcgagacccacgctcaccggctccagattta<br>tcagcaataaaccagccagccggaagggccgagcgcagaagtggtcctgcaactttatccgcctccatccagtctattaatt<br>gttgccgggaagctagagtaagtagttcgccagttaatagtttgcgcaacgttgttgccattgctacaggcatcgtggtgtcacg<br>ctcgtcgtttggtatggcttcattcagctccggttcccaacgatcaaggcgagttacatgatcccccatgttgtgcaaaaaagcg<br>gttagctccttcggtcctccgatcgttgtcagaagtaagttggccgcagtgttatcactcatggttatggcagcactgcataattctc<br>ttactgtcatgccatccgtaagatgcttttctgtgactggtgagtactcaaccaagtcattctgagaatagtgtatgcggcgaccg<br>agttgctcttgcccggcgtcaatacgggataataccgcgccacatagcagaactttaaaagtgctcatcattggaaaacgttctt<br>cggggcgaaaactctcaaggatcttaccgctgttgagatccagttcgatgtaacccactcgtgcacccaactgatcttcagcat<br>cttttactttcaccagcgtttctgggtgagcaaaaacaggaaggcaaaatgccgcaaaaaagggaataagggcgacacgg<br>aaatgttgaatactcatactcttcctttttcaatattattgaagcatttatcagggttattgtctcatgagcggatacatatttgaatgtat<br>ttagaaaaataaacaaataggggttccgcgcacatttccccatggtgccacctgacgtctaagaaaccattattatcatgacatt<br>aacctataaaaataggcgtatcacgaggcagaatttcagataaaaaaaatcct |
| Plasmid expressing chimeric P2-P1(S’) tail fiber and chaperone protein U (pP2-P1(S’)) |

(Benchling link: https://benchling.com/s/seq-FAG6ibePA6kzILw89oZQ?m=slm-sy1f4HfVnnIIMFe6hMJg)

tagctttcgctaaggatgatttctggaattcgcggccgcttctagagtacgctctccttgaatgttgtctggtagttctacaaatgaat
ccagatagcataacttttatatattgtgcaatctcacatgcatgagcataaaattcagaaccgttatcaccactgccggtgcagc
aaagctggcagcggcaaccgcgccgggagggcggaaggtcaacattaccacgatggccgtcggggatggcggtggtaa
attgcctgtcccggatgccggacagaccgggcttatccatgaagtctggcgacatgcgctgaacaaaatcagccaggacaa
acgaaacagtaattatattatcgccgagctggttattccgccggaggtgggcggtttctggatgcgtgagcttggcctgtacgat
gatgcgggaacgttaattgccgtggcgaacatggccgaaagctataagccagcccttgccgaaggctcaggacgttcgcag
acctgtcgcatggtcatcatcgtcagcagtgtggcctcagtggagctgaccattgacaccacaacggtgatggcgacgcagg
attacgttgatgacaaaattgcagagcacgaacagtcacgacgtcacccggacgcctcgctgacagcaaaaggttttactca
gttaagcagtgcgaccaacagcacgtctgaaacactggccgcaacgccgaaagcggtaaaggccgcgtatgacctggct
aacgggaaatataccgcacaggacgccaccacagcgcgaaaaggccttgtccagcttagtagcgccaccaacagcacgt
ctgaaacgctcgccgcaacaccaaaagccgttaagacggtaatggatgaaacgaacaaaaaagcgccattaaacagcc
ctgcactgaccggaacgccaacgacgccaactgcgcgacagggaacgaataatactcagatcgcaaacacggctttcgtt
atggccgcgattgccgcccttgtagactcgtcgcctgacgcactgaatacgctgaacgagctggcggcggcgctgggcaat
gacccgaattttgctaccaccatgactaatgcgcttgcgggtaagcaaccgaaagatgctaccctgacggcgctggcgggg
cttgctactgcggcagacaggtttccgtattttacggggaatgatgttgccagcctggcgaccctgacaaaagtcgggcggga
tattctggctaaatcgagcattcttgctgttatccaataccttggtttaagagaactcggtaccagcggtgaaaagatcccctgtt
gagcacggctaacacatggagtgcacgccagactttcaacggcgggatcaccggggcgctgacagggaacgccgatac
cgcaacgaaattgaaaacagccagaaacattaatggcgtcaggttcgatggttctggtgacattaatatcaatactctggtatc
gcgcggtcgcgtaacggccctggaggcgaatgcacagggaacatccgggattcagctgtatgaggcatacaacaatggct
acccttcccccctatggcaatgtgcttcaccttaaaggtgccaccgctgctggcgaaggtgagttattcattggctggagtggcac
gagcggtgcccatgcgcccgtacatatccgttcgcggcgggatactgattctgccaactggtctgaatgggcgcaggtctata
cgtcaaaagattcaattcccggcgtcaatgccaaaggggatcaggatacctctggtaatgcggctacagcgaccaagttgca
gacagcatgtactatcaacggtgtctcgtttgatggttctaaaaatattgagctaacggctgaaaatttaaatcttgagcgaaca
gtagaattagccgctgggtcattgcagaaaaatcagaacggcgcggatattcctggaaaagataccttcacaaaaaatattg
gtgcatgtcgcgcttttcacagttctattagtacaggtgcagggaactggacaacggcacaattgattgaatggctggattctca
aggggcattcaatcacccatactggatgtgcaaatgttcatggtcgtacggcaataataaaattataaccgatactggctgtgg
aactattcatcttgcaggttgcgttattgaggttatgggtaataaaggtgccatgaccatccgtgtaacaacaccaagcacttcc
agcggtggcggaatcactaacgctcaattcacttatattaatcatggtgatgcttacgctcctggctggcgacgagactacaac
acgaaaaacctgcaacctgcatttgctttagggcagacaggaaacagggttgcaaatgataaagctgttggctggaactgg
aatagcggtgtttatgatgcagacctaaaaggcgcatcaacattaattcttcatttcaatatgaacgcgggtagctgcccggctg
tacaattacgcgtgaattataagaacggcggtatttattatcgttcagcgcgtgatggttatggatttgaggctgactggtcagagt
tttacaccacaacccgcaaaccctctgcgggggatgttggtgcatatacgcaggcagaatgtaactcaaggtttattacaggt
attcgcctgggcggtctgtcatctgtccagacatggaatggccccggctggtctgacaggtcaggttatgtcgttacgggttcagt
taacgggaaccgtgatgaattaattgatacaacacaggcaaggccaattcagtattgcattaatgggacgtggtataacgcg
gggagtatttaattatgatgcacttaagaaatattacagctggcaaccctaaaacaaaagagcaataccagctaacgaaac
aatttaacatcaaatggctttatacagaggatggaaaaaactggtatgaggaacaaaagaatttccagtatgatacgttgaaa
atggcctatgaccacaacggcgttattatttgtattgaaaaggatgtttcagcaattaatccagaaggcgcaagcgtcgttgaat
tacctgatattacagcaaatcgccgggctgatatttctggtaaatggatgttcaaagatggcgtagtggtaaagcgaacttatac
cgaggaagagcagaggcaacaagcggaaaatgaaaagcaaagtctgctacagctcgtcagggataaaacccagctatg
ggactcacagctacggctgggtatcatttccgccgagaataagcagaaattaaccgagtggatgctctttgcgcagaaagtc
gaatccacagacacctccagcctaccagtaacatttcccgaacaacctgaatgagacaaggcaaggatccgaattcgagc
tccgtcgacaagcttgcggccgcactcgagcaccaccaccaccaccactgagatccggctgctaacaaagcccgaaagg
aagctgagttggctgctgccaccgctgagcaataactagcataaccccttggggcctctaaacgggtcttgaggggttttttgct
gaaaggaggaactatatccggattaataatactagtagcggccgctgcagtccggcaaaaaagggcaaggtgtcaccacc
ctgccctttttctttaaaaccgaaaagattacttcgcgttatgcaggcttcctcgctcactgactcgctgcgctcggtcgttcggctg
cggcgagcggtatcagctcactcaaaggcggtaatctcgaggttacattgtcgatctgttcatggtgaacagctttaaatgcac
caaaaactcgtaaaagctctgatgtatctatcttttttacaccgttttcatctgtgcatatggacagttttccctttgatatctaacggtg
aacagttgttctacttttgtttgttagtcttgatgcttcactgatagatacaagagccataagaacctcagatccttccgtatttagcc
agtatgttctctagtgtggttcgttgtttttgcgtgagccatgagaacgaaccattgagatcatgcttactttgcatgtcactcaaaaa
ttttgcctcaaaactggtgagctgaatttttgcagttaaagcatcgtgtagtgtttttcttagtccgttacgtaggtaggaatctgatgt
aatggttgttggtattttgtcaccattcatttttatctggttgttctcaagttcggttacgagatccatttgtctatctagttcaacttggaa
aatcaacgtatcagtcgggcggcctcgcttatcaaccaccaatttcatattgctgtaagtgtttaaatctttacttattggtttcaaaa

cccattggttaagccttttaaactcatggtagttattttcaagcattaacatgaacttaaattcatcaaggctaatctctatatttgcctt
gtgagttttcttttgtgttagttcttttaataaccactcataaatcctcatagagtatttgttttcaaaagacttaacatgttccagattata
ttttatgaatttttttaactggaaaagataaggcaatatctcttcactaaaaactaattctaatttttcgcttgagaacttggcatagttt
gtccactggaaaatctcaaagcctttaaccaaaggattcctgatttccacagttctcgtcatcagctctctggttgctttagctaata
caccataagcattttccctactgatgttcatcatctgagcgtattggttataagtgaacgataccgtccgttctttccttgtagggttttc
aatcgtggggttgagtagtgccacacagcataaaattagcttggtttcatgctccgttaagtcatagcgactaatcgctagttcatt
tgctttgaaaacaactaattcagacatacatctcaattggtctaggtgattttaatcactataccaattgagatgggctagtcaatg
ataattacatgtccttttcctttgagttgtgggtatctgtaaattctgctagacctttgctggaaaacttgtaaattctgctagaccctct
gtaaattccgctagacctttgtgtgttttttttgtttatattcaagtggttataatttatagaataaagaaagaataaaaaaagataaa
aagaatagatcccagccctgtgtataactcactactttagtcagttccgcagtattacaaaaggatgtcgcaaacgctgtttgctc
ctctacaaaacagaccttaaaaccctaaaggcttaagtagcaccctcgcaagctcgggcaaatcgctgaatattccttttgtct
ccgaccatcaggcacctgagtcgctgtctttttcgtgacattcagttcgctgcgctcacggctctggcagtgaatgggggtaaat
ggcactacaggcgccttttatggattcatgcaaggaaactacccataatacaagaaaagcccgtcacgggcttctcagggcg
ttttatggcgggtctgctatgtggtgctatctgactttttgctgttcagcagttcctgccctctgattttccagtctgaccacttcggattat
cccgtgacaggtcattcagactggctaatgcacccagtaaggcagcggtatcatcaacaggcttacccgtcttactgtccctag
tgcttggattctcaccaataaaaaacgcccggcggcaaccgagcgttctgaacaaatccagatggagttctgaggtcattact
ggatctatcaacaggagtccaagcgagctcgtaaacttggtctgacagttaccaatgcttaatcagtgaggcacctatctcagc
gatctgtctatttcgttcatccatagttgcctgactccccgtcgtgtagataactacgatacgggagggcttaccatctggcccca
gtgctgcaatgataccgcgagacccacgctcaccggctccagatttatcagcaataaaccagccagccggaagggccgag
cgcagaagtggtcctgcaactttatccgcctccatccagtctattaattgttgccgggaagctagagtaagtagttcgccagttaa
tagtttgcgcaacgttgttgccattgctacaggcatcgtggtgtcacgctcgtcgtttggtatggcttcattcagctccggttcccaa
cgatcaaggcgagttacatgatcccccatgttgtgcaaaaaagcggttagctccttcggtcctccgatcgttgtcagaagtaagt
tggccgcagtgttatcactcatggttatggcagcactgcataattctcttactgtcatgccatccgtaagatgcttttctgtgactggt
gagtactcaaccaagtcattctgagaatagtgtatgcggcgaccgagttgctcttgcccggcgtcaatacgggataataccgc
gccacatagcagaactttaaaagtgctcatcattggaaaacgttcttcggggcgaaaactctcaaggatcttaccgctgttgag
atccagttcgatgtaacccactcgtgcacccaactgatcttcagcatcttttactttcaccagcgtttctgggtgagcaaaaacag
gaaggcaaaatgccgcaaaaaagggaataagggcgacacggaaatgttgaatactcatactcttcctttttcaatattattga
agcatttatcagggttattgtctcatgagcggatacatatttgaatgtatttagaaaaataaacaaataggggttccgcgcacattt
cccatggtgccacctgacgtctaagaaaccattattatcatgacattaacctataaaaataggcgtatcacgaggcagaatttc
agataaaaaaaaatcct

Plasmid expressing chimeric P2-ϕV10 tail fiber (pP2-phiV10)
(Benchling link: https://benchling.com/s/seq-ZFEaqfjbIqOaGLFpmy7a?m=slm-taXpYJ5d2uDXPcT0XPWZ)

tagctttcgctaaggatgatttctggaattcgcggccgcttctagagtacgctctccttgaatgttgtctggtagttctacaaatgaat
ccagatagcataacttttatatattgtgcaatctcacatgcatgagcataaaattcagaaccgttatcaccactgccggtgcagc
aaagctggcagcggcaaccgcgccgggagggcggaaggtcaacattaccacgatggccgtcggggatggcggtggtaa
attgcctgtcccggatgccggacagaccgggcttatccatgaagtctggcgacatgcgctgaacaaaatcagccaggacaa
acgaaacagtaattatattatcgccgagctggttattccgccggaggtgggcggtttctggatgcgtgagcttggcctgtacgat
gatgcgggaacgttaattgccgtggcgaacatggccgaaagctataagccagcccttgccgaaggctcaggacgttcgcag
acctgtcgcatggtcatcatcgtcagcagtgtggcctcagtggagctgaccattgacaccacaacggtgatggcgacgcagg
attacgttgatgacaaaattttagcggcaaatgatggatttaagtttattggtcagtgccctgatatattgacactaagaacaatag
aacctgaaaaaaatggtcaaaggatcacgttgcgacagcatacgatcggaactgggcttggtggaggggtattcagagctg
tacttgatggaactggctataccgatgatgatggtgttgtaataaaaacagcaggtggttctgtatggttacgggttaatgcagat
aaggttaatccgtttatgtttggggccactggcgtggctgatgatacagcagcactgcaaaagatgcttgaatgtggcagggc
ggctgagcttggcacaaatgtctggaaagcatctaatcttgagctaaataacaagtcgtgttcactatctgggtctggtctacac
gtttccaggatagagcaaatatctggggcaacaggcgcgttactgacaattactcaggattgcagcctgatttatttgtccgattg
tggcctgtatggggatggaataactgcgggaacaagcggggtaacgatggagacaggaaacccaggtggggcaccaag
ctatccattcaataccgcccctgacgttcgtagggatctttacattagcaatgttcacattacaggatttgacgagttaggatttgat
tatcctgaaacgaatttttccgtctcaacgcatggattattcataagaaacattaaaaaaaccggggctaagatcggaactacc
gattttacatggacaaaccttcaaattgatacgtgtgggcaggaatgcttagtgcttgatggtgctggtaattgcagaattataggt
gccaaattaatttgggctggttcagaaaatgaaacaccatattcaggattacgtatttctaattcacaaaatgtaaacatgacag
gagttgagttacaggactgtgcatacgatggattgtatattaaaaattcaactgttgctatcagtgggttgaatactaacagaaat
agtgcatcatcaaatctgtcataccataatatggtttttgaaaacagtatagtaactgtagatggttatgtttgccgtaactatgctg
ctacatcgctatatgatctaaattcacaagccggaaatgtgaggtgtattggttccgatagtaccgtattgataaatggtatttacg

agtcagaagtaaacagcgaacgactcatgggtgacaataatcttattcagccatattctggtgatttaataataaatggcctaa
aaaactattatacatatacaggaagcgtaaaaaacaatattcctacatttgacggagtcgttacaaccgcaacatatgtaagc
gctccttcgatactagggcaaggaaacatgcttaagttaacgcaatcaaacaaggataagttattattttctgataaagtatcga
gacatgggtgtacaatcggccttgttctaatcccgtcatttacgggtgcaacaactatgacagctttcactctggggagtggatat
tcgccctctggaaactctgctgttatgcaatttatagttaactccagtggtgtgcagactatagcaatattattatccggtgatggtat
aactcaaacgttaacaagcgatctgacaacggagcaagcgttagcttcaggaggcgtatatcattttgcgatgggattcgctc
caggccgattatggtggtctattattgatataaataccggaaggcgtatacgacgggcgtacagacaaccagatcttcacgcc
gcatttaatagcatattcaacagcggaacatcttctatcaccgcattttcaggaccattagcaggtgatattgcatgtgaagggg
caggttcgcatgtttatgttggcggtttctcatctgagtcagactacgcagcgagcagaatgtacggattatttaccccagtagac
ctggataaacaatactcattccgcacgctgaacggaaatatttaaggatccgaattcgagctccgtcgacaagcttgcggccg
cactcgagcaccaccaccaccaccactgagatccggctgctaacaaagcccgaaaggaagctgagttggctgctgccac
cgctgagcaataactagcataaccccttggggcctctaaacgggtcttgaggggttttttgctgaaaggaggaactatatccgg
attaataatactagtagcggccgctgcagtccggcaaaaaagggcaaggtgtcaccaccctgcccttttttctttaaaaccgaa
aagattacttcgcgttatgcaggcttcctcgctcactgactcgctgcgctcggtcgttcggctgcggcgagcggtatcagctcact
caaaggcggtaatctcgaggttacattgtcgatctgttcatggtgaacagctttaaatgcaccaaaaactcgtaaaagctctgat
gtatctatcttttttacaccgttttcatctgtgcatatggacagttttccctttgatatctaacggtgaacagttgttctacttttgtttgttagt
cttgatgcttcactgatagatacaagagccataagaacctcagatccttccgtatttagccagtatgttctctagtgtggttcgttgtt
tttgcgtgagccatgagaacgaaccattgagatcatgcttactttgcatgtcactcaaaaattttgcctcaaaactggtgagctga
atttttgcagttaaagcatcgtgtagtgtttttcttagtccgttacgtaggtaggaatctgatgtaatggttgttggtattttgtcaccattc
atttttatctggttgttctcaagttcggttacgagatccatttgtctatctagttcaacttggaaaatcaacgtatcagtcgggcggcct
cgcttatcaaccaccaatttcatattgctgtaagtgtttaaatctttacttattggtttcaaaacccattggttaagccttttaaactcat
ggtagttattttcaagcattaacatgaacttaaattcatcaaggctaatctctatatttgccttgtgagttttcttttgtgttagttcttttaat
aaccactcataaatcctcatagagtatttgttttcaaaagacttaacatgttccagattatattttatgaattttttaactggaaaaga
taaggcaatatctcttcactaaaaactaattctaatttttcgcttgagaacttggcatagtttgtccactggaaaatctcaaagccttt
aaccaaaggattcctgatttccacagttctcgtcatcagctctctggttgctttagctaatacaccataagcattttccctactgatgt
tcatcatctgagcgtattggttataagtgaacgataccgtccgttctttccttgtagggttttcaatcgtggggttgagtagtgccaca
cagcataaaattagcttggtttcatgctccgttaagtcatagcgactaatcgctagttcatttgctttgaaaacaactaattcagac
atacatctcaattggtctaggtgattttaatcactataccaattgagatgggctagtcaatgataattacatgtccttttcctttgagtt
gtgggtatctgtaaattctgctagacctttgctggaaaacttgtaaattctgctagaccctctgtaaattccgctagacctttgtgtgtt
ttttttgtttatattcaagtggttataatttatagaataaagaaagaataaaaaaagataaaaagaatagatcccagccctgtgtat
aactcactactttagtcagttccgcagtattacaaaaggatgtcgcaaacgctgtttgctcctctacaaaacagaccttaaaacc
ctaaaggcttaagtagcaccctcgcaagctcgggcaaatcgctgaatattccttttgtctccgaccatcaggcacctgagtcgct
gtctttttcgtgacattcagttcgctgcgctcacggctctggcagtgaatgggggtaaatggcactacaggcgccttttatggattc
atgcaaggaaactacccataatacaagaaaagcccgtcacgggcttctcagggcgttttatggcgggtctgctatgtggtgct
atctgactttttgctgttcagcagttcctgccctctgattttccagtctgaccacttcggattatcccgtgacaggtcattcagactggc
taatgcacccagtaaggcagcggtatcatcaacaggcttacccgtcttactgtccctagtgcttggattctcaccaataaaaaa
cgcccggcggcaaccgagcgttctgaacaaatccagatggagttctgaggtcattactggatctatcaacaggagtccaagc
gagctcgtaaacttggtctgacagttaccaatgcttaatcagtgaggcacctatctcagcgatctgtctatttcgttcatccatagtt
gcctgactccccgtcgtgtagataactacgatacgggagggcttaccatctggccccagtgctgcaatgataccgcgagacc
cacgctcaccggctccagatttatcagcaataaaccagccagccggaagggccgagcgcagaagtggtcctgcaactttat
ccgcctccatccagtctattaattgttgccgggaagctagagtaagtagttcgccagttaatagtttgcgcaacgttgttgccattg
ctacaggcatcgtggtgtcacgctcgtcgtttggtatggcttcattcagctccggttcccaacgatcaaggcgagttacatgatcc
cccatgttgtgcaaaaaagcggttagctccttcggtcctccgatcgttgtcagaagtaagttggccgcagtgttatcactcatggtt
atggcagcactgcataattctcttactgtcatgccatccgtaagatgcttttctgtgactggtgagtactcaaccaagtcattctgag
aatagtgtatgcggcgaccgagttgctcttgcccggcgtcaatacgggataataccgcgccacatagcagaactttaaaagt
gctcatcattggaaaacgttcttcggggcgaaaactctcaaggatcttaccgctgttgagatccagttcgatgtaacccactcgt
gcacccaactgatcttcagcatcttttactttcaccagcgtttctgggtgagcaaaaacaggaaggcaaaatgccgcaaaaaa
gggaataagggcgacacggaaatgttgaatactcatactcttcctttttcaatattattgaagcatttatcagggttattgtctcatg
agcggatacatatttgaatgtatttagaaaaataaacaaataggggttccgcgcacatttcccatggtgccacctgacgtctaa
gaaaccattattatcatgacattaacctataaaaataggcgtatcacgaggcagaatttcagataaaaaaaatcct

## References

(1) Tridgett, M., Ababi, M., Osgerby, A., Ramirez Garcia, R., and Jaramillo, A. (2021) Engineering bacteria to produce pure phage-like particles for gene delivery. *ACS Synth. Biol. 10*, 107–114.

(2) Wei, J., Goldberg, M. B., Burland, V., Venkatesan, M. M., Deng, W., Fournier, G., Mayhew, G. F., Plunkett, G., Rose, D. J., Darling, A., Mau, B., Perna, N. T., Payne, S. M., Runyen-Janecky, L. J., Zhou, S., Schwartz, D. C., and Blattner, F. R. (2003) Complete genome sequence and comparative genomics of *Shigella flexneri* serotype 2a strain 2457T. *Infect. Immun. 71*, 2775–2786.

(3) Mostowy, S., Bonazzi, M., Hamon, M. A., Tham, T. N., Mallet, A., Lelek, M., Gouin, E., Demangel, C., Brosch, R., Zimmer, C., Sartori, A., Kinoshita, M., Lecuit, M., and Cossart, P. (2010) Entrapment of intracytosolic bacteria by septin cage-like structures. *Cell Host Microbe 8*, 433–444.

(4) Wolfson, E. B., Elvidge, J., Tahoun, A., Gillespie, T., Mantell, J., McAteer, S. P., Rossez, Y., Paxton, E., Lane, F., Shaw, D. J., Gill, A. C., Stevens, J., Verkade, P., Blocker, A., Mahajan, A., and Gally, D. L. (2020) The interaction of *Escherichia coli* O157 :H7 and *Salmonella* Typhimurium flagella with host cell membranes and cytoskeletal components. *Microbiology 166*, 947–965.

(5) Datsenko, K. A., and Wanner, B. L. (2000) One-step inactivation of chromosomal genes in *Escherichia coli* K-12 using PCR products. *Proc. Natl. Acad. Sci. USA 97*, 6640–6645.
